# Modulation of comorbid depression of neuropathic pain by dopamine input from VTA to the ventral hippocampus

**DOI:** 10.7150/thno.104394

**Published:** 2025-03-10

**Authors:** Yuanyuan Ji, Yifan Xiao, Xiaoyan Bai, Junxiang Gu, Teng Ma, Yue Feng, Jian Wang, Yupeng Feng, Tao Chen, Jianghua Lai, Juan Shi, Jinlian Li

**Affiliations:** 1 Department of Anatomy, School of Medicine, Northwest University, Xi'an 710069, China.; 2 College of Forensic Science, Key Laboratory of National Health Commission for Forensic Science, National Biosafety Evidence Foundation, Xi'an Jiaotong University, Xi'an 710061, China.; 3 Department of Human Anatomy, Histology and Embryology & K. K. Leung Brain Research Centre, Preclinical School of Medicine, Fourth Military Medical University, Xi'an 710032, China.; 4 Functional and Molecular Imaging Key Lab of Shaanxi Province, Department of Radiology, Tangdu Hospital, Fourth Military Medical University, Xi'an 710038, China.; 5 Department of Neurosurgery, the Second Affiliated Hospital of Xi'an Jiaotong University, Xi'an 710004, China.; 6 Department of Neurosurgery, Tangdu Hospital, Fourth Military Medical University, Xi'an 710038, China.

**Keywords:** ventral tegmental area, ventral hippocampus, depression, chronic pain, dopamine receptor

## Abstract

**Background:** Chronic pain syndrome is a devastating disorder with poor clinical treatment. The circuitry and molecular mechanisms for depression comorbid with chronic pain are thus far unclear. We characterized the projection from the ventral tegmental area (VTA) to the ventral hippocampus (vHPC) and assessed the functional significance of the pathway in chronic pain-induced depressive comorbidity.

**Methods:** A neuropathic cuff model was adopted and discriminated against the susceptible and resilient groups with or without depression-like behaviors, respectively. The anatomical feature and function of the VTA-vHPC pathway were assessed by tracer and virus-based tracing, immunofluorescent staining, fluorescence *in situ* hybridization (FISH), designer receptors exclusively activated by designer drugs (DREADDs), optogenetics, and *ex vivo* electrophysiology.

**Results:** A group of medially-located dopaminergic (DAergic) neurons displayed few overlappings with the medial prefrontal cortex- or nucleus accumbens-projecting neurons, constituting the major projection from the VTA to the vHPC. The activity of vHPC-projecting DAergic neurons was downregulated in the susceptible group but not in the resilient group, as manifested by the decreased expression of tyrosine hydroxylase (TH), TH/FOS double-labeling, and excitability in retrogradely labeled VTA neurons. Chemogenetic activation of the pathway significantly improved depression rather than pain phenotype, but caspase 3-based ablation induced depression. Optogenetic activation of the VTA^DA^-vHPC pathway produced similar anti-depressant effects in cuff animals in an equally D1 or D2 receptor-dependent manner. FISH and Western blotting disclosed a low-segregated expression of the D1 receptor in the pyramidal neurons and a highly-segregated expression of the D2 receptor in GABAergic neurons in vHPC, which underwent no change or upregulation following cuffing.

**Conclusion:** Our results demonstrate a medially dominant VTA^DA^ projection to the vHPC. Reinforcement of this pathway can reverse the depression without affecting pain, thus providing insights into a connectivity-based strategy in the treatment of comorbid depression.

## Introduction

Chronic pain is a debilitating and deleterious disorder with a significant socioeconomic impact 1. The prevalence of chronic pain has increased worldwide, affecting approximately 20% of the world population 2. The related healthcare costs are between $560 and $635 billion annually in the US 3. Nonetheless, the clinical treatment for chronic pain is far from satisfactory, probably because the symptomatology of chronic pain is complex and multi-dimensional 1, 3. In addition to sensory hypersensitivity, chronic pain is also characterized by affective and/or cognitive symptoms 2, 4. Depression is the most frequently encountered comorbidity and is likely to be present in 40-50% of chronic pain patients 5. In other words, nearly half of the patients with chronic pain suffer from devastating mood disorders. Even worse, chronic pain and depression can interact and exacerbate one another (pain-depression dyad), making the treatment more challenging 4. Thus, unraveling the circuitry and molecular changes induced by chronic pain and its comorbidity, especially through converging the overlapping brain regions and pathways, will pave the way for developing refined and effective treatments.

Dopaminergic (DAergic) neurons in the ventral tegmental area (VTA) play critical roles in mediating reward-related and goal-directed behaviors 6. Emerging evidence also points to an important function of the neurons in the perception and modulation of chronic pain symptoms 4. The mesolimbic projection to the nucleus accumbens (NAc) forms one of the major outputs of the VTA. Research has shown that the VTA-NAc pathway is maladaptive in chronic pain and/or depression and constitutes one of the shared underpinnings for chronic pain symptoms 4. For instance, human and animals with chronic pain showed decreased dopamine (DA) release in the NAc, and optogenetic activation of the VTA DAergic (VTA^DA^) neurons that project to the NAc produced analgesic effects in neuropathic and cancer pain 7-9. Ren *et al.* reported that spared nerve injury can cause a reduction in the spontaneous spiking of VTA^DA^ neurons projecting to the medial shell of NAc (NAcMed) and an elevated excitability of indirect spiny neurons in the NAcMed 10. Chemogenetic excitation of the indirect spiny neurons worsens tactile allodynia, whereas inhibition alleviates allodynia 10. Further, a recent study demonstrated that a glutamatergic pathway from the dorsal raphe nucleus (DRN^Glu^) to VTA^DA^ neurons is dampened during neuropathic pain, and that optogenetic stimulation of the DRN^Glu^-VTA^DA^ circuit alleviates pain and comorbid anhedonia via D2 and D1 dopamine receptors in NAcMed, respectively 11. These results support the important role of the VTA^DA^-NAc pathway in modulating the sensory and affective symptoms of chronic pain.

Besides the NAc, VTA DAergic neurons also target the prefrontal cortex (PFC), anterior cingulate cortex (ACC), amygdala, hippocampus, ventral pallidum, periaqueductal grey, bed nucleus of the stria terminalis (BNST), olfactory tubercle, and locus coeruleus (LC) 4, 6. Identifying the projection neurons to these areas and the functional evaluation of pathways remain scant. The hippocampus is another important limbic brain region involved in pain and emotion processing and shows functional segregation along the septo-temporal (dorsal-ventral) axis 12, 13. Generally, the dorsal hippocampus (dHPC, posterior in primates) primarily correlates with cognitive processing, while the ventral hippocampus (vHPC, anterior in primates) is more related to emotional regulation 12, 14. Earlier tracing studies reported that the hippocampus, especially the vHPC is innervated by the DAergic projection from the VTA 15. Immunohistochemical staining also demonstrated the existence of tyrosine hydroxylase (TH)-positive terminals in the vHPC in noradrenaline-depleted rats 16. Moreover, neurons in the vHPC express dopamine receptors, which have been demonstrated to play important roles in modulating synaptic plasticity and regulating pathophysiological functions such as working memory 17, 18. However, thus far, the feature of an anatomical connection between the VTA and the vHPC and the functional significance of the pathway in chronic pain is still unclear.

In this study, we defined the anatomical properties of the VTA-vHPC pathway using a combination of traditional and virus-based tracing, examined the adaptive changes of the projection neurons by discriminating the sole pain from the comorbid depression, assessed the function of the pathway using chemogenetics, caspase 3-based ablation, and optogenetics, and explored the underlying receptor mechanism.

## Results

### Pathway of DAergic neurons in the medial VTA projecting to the vHPC

**Figure 1:**
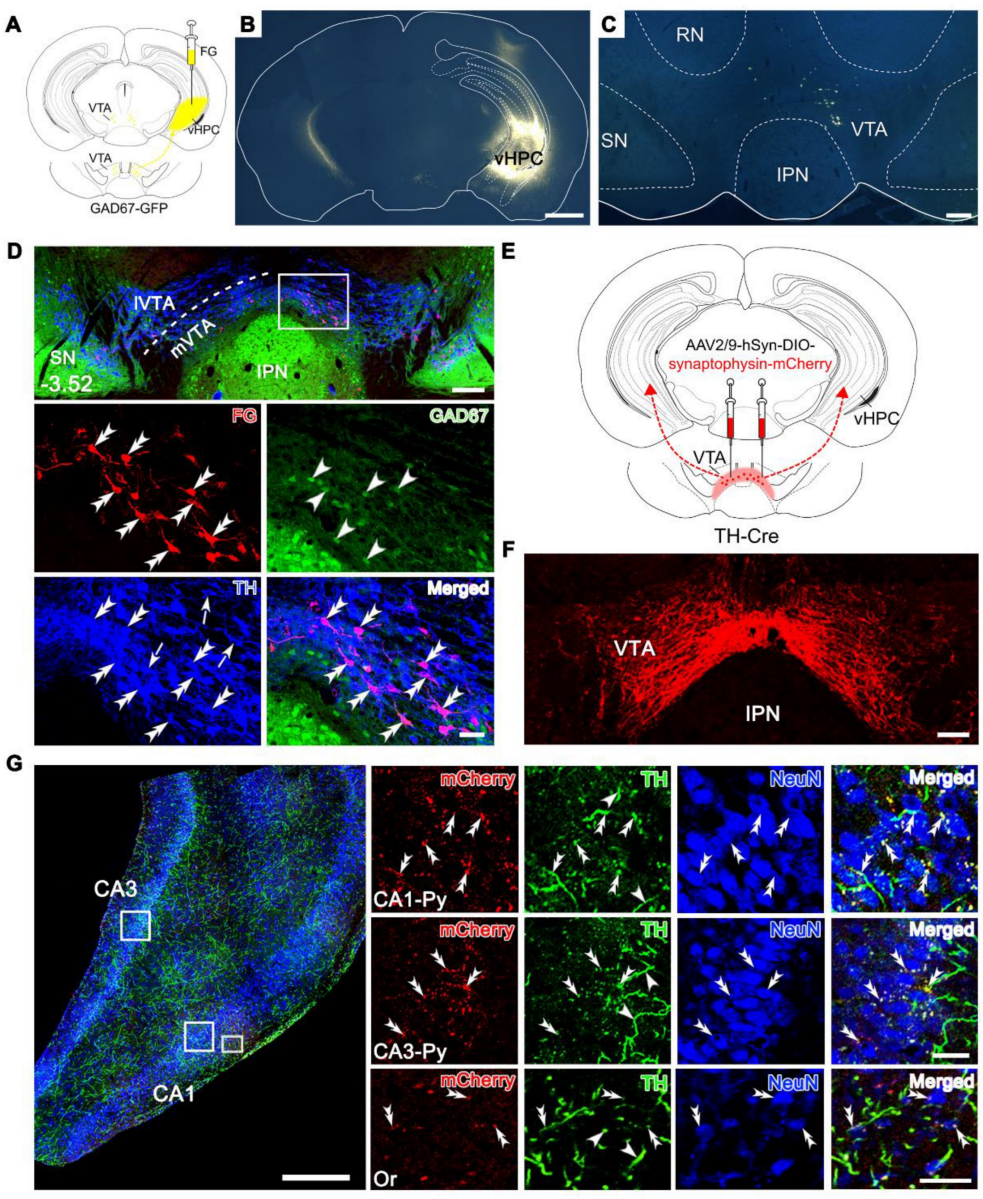
** Projection from the dopaminergic neurons in the VTA to the vHPC. (A)** Schematic diagram for retrograde tracing of the VTA-vHPC pathway by injecting FG into the right vHPC in GAD67-GFP mice. **(B)** Representative coronal section showing the FG injection site. Scale bar: 1 mm. **(C)** Images showing the distribution of FG-labeled neurons in the VTA, mainly on the ipsilateral side. Scale bar: 200 μm. **(D)** Triple immunofluorescent illustration of FG-labeled vHPC-projecting neurons (red), GFP-labeled GABAergic neurons (green), and TH-labeled DAergic neurons (blue) in the middle VTA (Bregma: -3.52 mm), showing the distribution of FG in TH-positive but not GAD67-positive neurons. The dash line indicates the position of mVTA and lVTA. The area depicted by the white rectangle is enlarged in the lower two panels, with double arrowheads indicating FG/TH double-labeled neurons; arrowheads, GFP single-labeled neurons; arrows, TH single-labeled neurons. Scale bar: 200 μm (top panel), 50 μm (the lower two panels). **(E)** Schematic diagram of anterograde tracing of the VTA-vHPC pathway by injecting AAV2/9-hSyn-DIO-synaptophysin-mCherry into the bilateral VTA in TH-Cre mice. **(F)** Representative image for virus infection in the VTA. Scale bar: 100 μm. **(G)** Triple immunofluorescent illustration of synaptophysin-mCherry-labeled terminals (red) from the VTA, TH-positive fibers and terminals (green), and NeuN-positive cells (blue) in the vHPC. Rectangular areas in the pyramidal layers (Py) of CA1 and CA3, and the oriens layer of CA1 (Or) are amplified in the right panels, with double arrowheads indicating TH/synaptophysin-mCherry double-labeled terminals near neuronal cell bodies, and arrowheads indicating TH single-labeled fibers. Scale bar: 500 μm (left), 50 μm (right). IPN, interpeduncular nucleus; RN, red nucleus; SN, substantia nigra.

We investigated the anatomical connection from the VTA to the vHPC by injecting the retrograde tracer Fluoro-Gold (FG) into the right vHPC of mice expressing green fluorescent protein (GFP) under the glutamate decarboxylase (GAD) promoter (GAD67-GFP mice), i.e., mice specifically producing GFP in the GABA (γ-aminobutyric acid)-ergic neurons. The FG injection site was characterized by a dense core in the vHPC surrounded by a diffuse halo of the tracer (Figure 1A-B). A significant number of FG-labeled neurons was observed in the whole architecture of the VTA in an ipsilaterally dominant manner (ipsilateral: 80.2% ± 1.20%; contralateral: 19.8% ± 1.20%; Figure 1C-D, Figure S1, and Table S1). VTA can be divided into the medial (mVTA) and lateral (lVTA) parts, which show differential connectivity and pathophysiological responsivity to stimuli such as reward and aversion 19-21. We defined the location of the FG-labeled neurons along the rostrocaudal axis of the VTA and found that 79% of the projection neurons were located in the mVTA (79.0% ± 0.60%; Table S1). At the rostral level (Bregma -3.08), dense FG-labeled neurons were distributed in the lateral domain of the mVTA (near the substantia nigra) (Figure S1A), while at the rostral-middle level (Bregma -3.28), FG-labeled neurons could be seen in a mediolateral mixture in the mVTA (Figure S1B). At the middle to caudal levels (Bregma -3.52, -3.64, -3.80), most of the FG-labeled neurons were medially located in the mVTA (near the midline) (Figure 1C-D, Figure S1C-D, and Table S1).

VTA contains heterogeneous cell populations, including around 65% DAergic, 30% GABAergic, and 5% glutamatergic neurons 22. To clarify the type of VTA neurons projecting to the vHPC, we performed double immunofluorescent staining for FG and TH (for DAergic neurons) in the GAD67-GFP mice injected with FG in the vHPC. The results showed that FG-labeled neurons in the VTA were dominantly TH-immunoreactive (91.7% ± 1.49%; Figure 1D and Figure S1) but not GFP-positive and accounted for 12.7% ± 0.45% of all DAergic neurons. Consistently, 78.6% ± 1.04% of FG/TH double-labeled neurons were distributed in the mVTA (Table S1). To confirm the DAergic projection from the VTA to the vHPC, we injected anterograde adeno-associated virus (AAV) carrying Cre-dependent synaptophysin-mCherry fusion sequence (AAV2/9-hSyn-DIO-synaptophysin-mCherry) into the bilateral VTA of TH-Cre mice (Figure 1E-F). After 4 weeks, mCherry-expressing terminals were observed in the CA1 and CA3 regions of vHPC, which exhibited the appearance of puncta or boutons characterizing the synaptophysin location in presynaptic elements (Figure 1G). The terminals were relatively densely distributed in the pyramidal layer of the CA1 and CA3 and showed marked double-labeling with TH immunoreactivities around the NeuN-positive cell bodies (Figure 1G, upper and middle panels). Sparse puncta were also found in the vicinity of neurons in other laminae such as stratum oriens (Or, Figure 1G, lower panel). This reciprocal tracing indicated the existence of a dominant innervation from the mVTA DAergic neurons (mVTA^DA^) to the vHPC, possibly targeting pyramidal neurons and interneurons.

VTA is known for its mesolimbic projections, such as NAc and the medial prefrontal cortex (mPFC) 4. To further define the anatomical feature of the VTA-vHPC projection with these pathways, we injected FG, retrograde AAV expressing red fluorescent protein (AAV2/R-hSyn-mCherry) or enhanced green fluorescent protein (AAV2/R-hSyn-EGFP) into the vHPC, mPFC, and NAc, respectively, of normal C57BL/6J mice (Figure S2A-D). We found that mCherry-positive neurons targeting the mPFC tended to aggregate in the rostral and middle segments of the VTA, with both medially and laterally located features (Figure S2E-I). Comparatively, EGFP-positive neurons innervating the NAc were robustly distributed along most segments of the VTA (Figure S2E-I). Interestingly, many mCherry and EGFP double-labeled neurons were observed, which accounted for 60.2% ± 3.99% of mCherry-labeled neurons and 5.9% ± 1.04% of EGFP-labeled neurons. By contrast, we seldom detected FG and virus co-labeled neurons in the VTA. These results demonstrated that most of the vHPC-projecting VTA neurons appear not to send collaterals to the mPFC and NAc, thus may representing a unique cellular population thus far known.

### Classification of cuff mice into resilient and susceptible groups

**Figure 2:**
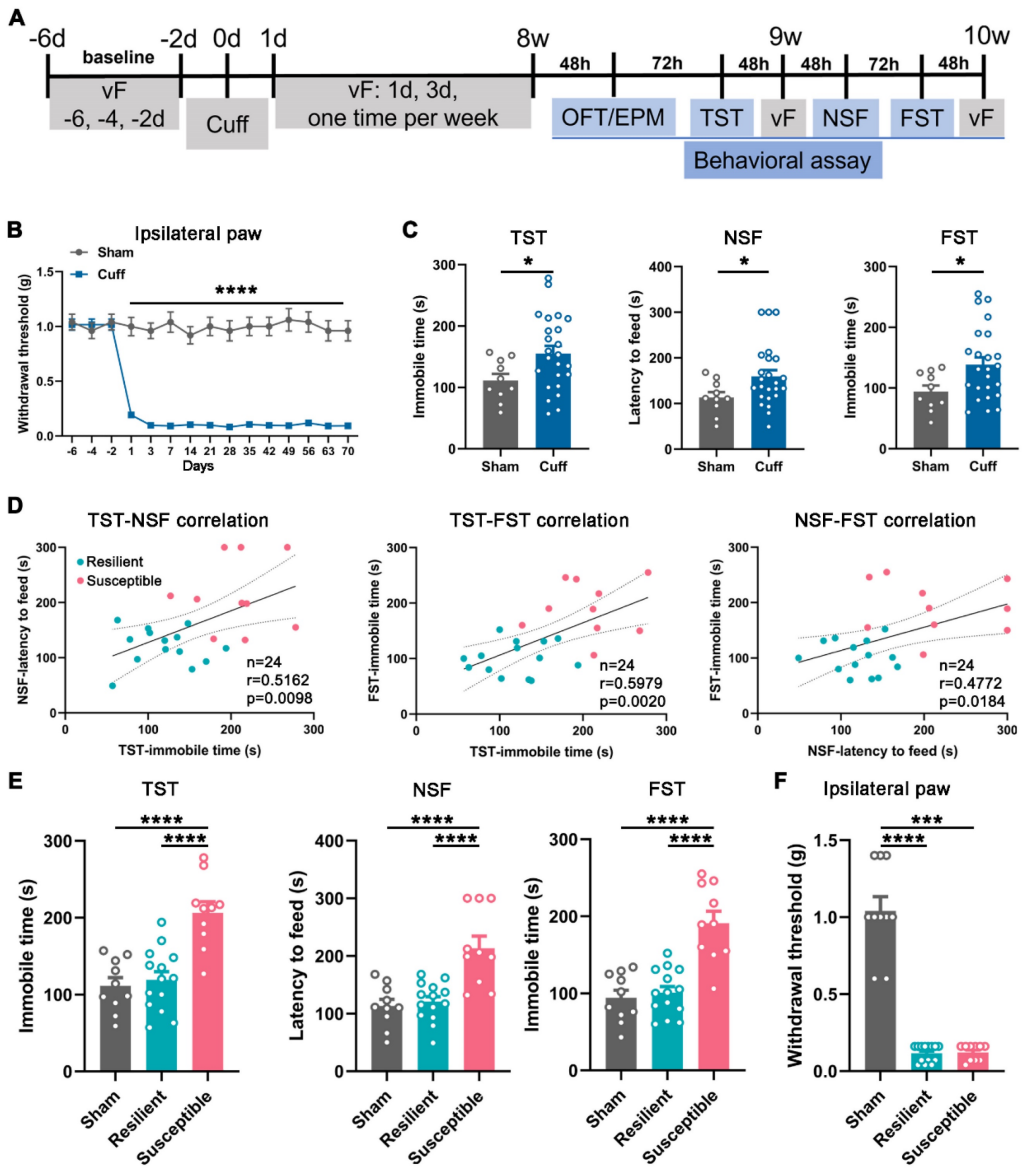
** Behavioral manifestations of the cuff mice and animal classification. (A)** Timeline for operation and behavioral testing. **(B)** Mechanical hypersensitivity of cuff mice in the ipsilateral hind paw. *n* = 10 in the sham group and 24 in the cuff group. ****P < 0.0001, by 2-way RM ANOVA with Šidák's post hoc test. **(C)** Mice with 8-week nerve cuffing showed increased immobile time in the TST and FST and increased latency to feed in the NSF. *n* = 10 in sham and 24 in cuff. *P < 0.05, TST and FST: unpaired Student's *t*-test; NSF: Mann-Whitney test. **(D)** Correlation analysis between every two performances in the TST, NSF, and FST in the cuff mice.* n* = 14 in the resilient group, *n* = 10 in the susceptible group. Pearson's correlation test. **(E)** Re-analysis of the animal performance in the TST, NSF, and FST according to the classification in D. Susceptible but not resilient mice showed prominently increased immobile time in the TST and FST and enhanced latency to feed in the NSF compared with sham mice. The data of sham group are the same as Figure 2C for making comparison. *n* = 10-14. ****P < 0.0001, by 1-way ANOVA with Tukey's post hoc test. **(F)** Resilient and susceptible mice showed identical mechanical hypersensitivity after 8-week cuffing. *n* = 10-14. ***P < 0.001, ****P < 0.0001, by Kruskal-Wallis test with Dunn's post hoc test.

Chronic pain is frequently comorbid with mood disorders 5, 23. Next, we employed a sciatic nerve cuff model and examined the animal behaviors (Figure 2A). Mice subjected to cuffing exhibited sustained mechanical allodynia in the ipsilateral but not the contralateral hind paw since the postoperative day (POD) 1 (Figure 2B and Figure S3A). In addition, at postoperative week (POW) 8 to 10, the cuff mice showed significantly increased latency to feed in the novelty suppressed feeding test (NSF) and elongated immobile time in the tail suspension test (TST) and forced swim test (FST) compared with the sham mice, manifesting the appearance of depression-like behaviors (Figure 2C). Nevertheless, the performance of cuff mice in the three tests was disparate, with some far different, whereas others almost nondifferential from the sham mice, indicating the diversity of individual sensitivity to the mood disorder induced by neuropathic pain (Figure 2C).

The K-means clustering approach is an unsupervised machine learning algorithm for clustering data into distinct groups based on similarities. We adopted this method to divide the cuff mice into the susceptible or resilient group according to the similarity of their performance in the TST, NSF, and FST (normalized as features). Subsequently, the correlation analysis between individual performances in every two tests was conducted. As shown in Figure 2D, the cuff mice exhibited consistent correlations between the two values in NSF, TST, and FST, suggesting the method's validity in discriminating the depressive phenotypes. Accordingly, re-analysis of the data demonstrated significant differences between the susceptible and resilient mice in the depressive performance (Figure 2E) but not in the pain behavior or anxiety sensitivity evaluated by the open field test (OFT) and elevated plus maze (EPM) (Figure 2F and Figure S3B-E), suggesting that the cuff mice at POW 8-10 indeed had two phenotypes, i.e., the comorbid depression and the 'sole' pain. The proportion of the susceptible mice over the total operated ones was 41.7% (10/24).

### Hypoactivity of vHPC-projecting VTA neurons in the susceptible group

**Figure 3:**
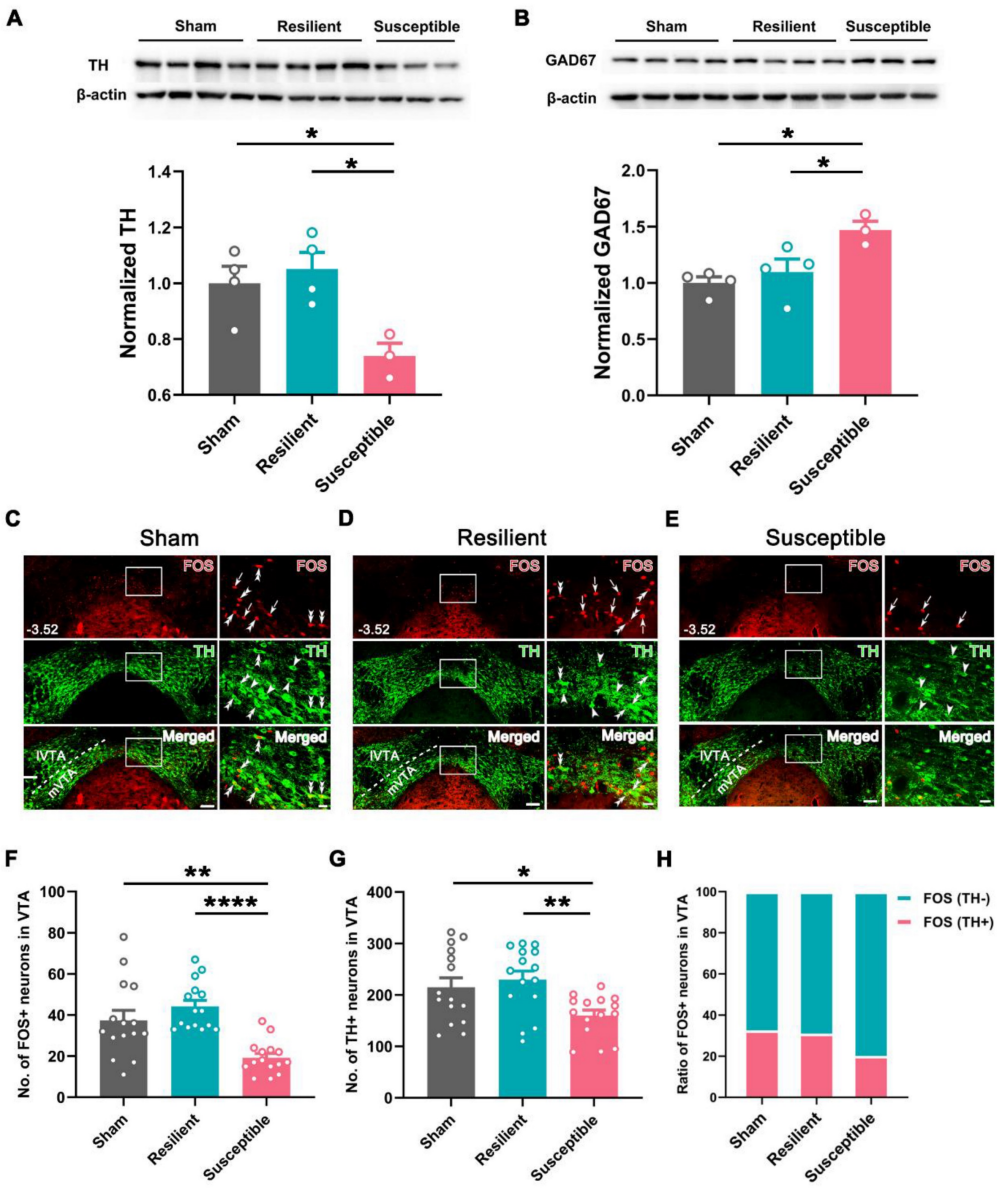
** Decreased activity in DAergic neurons and increased activity in GABAergic neurons in the VTA of susceptible mice. (A** and **B)** Western blotting of TH and GAD67 levels in VTA, showing decreased expression of TH and increased expression of GAD67 in the susceptible mice at POW 10. *n* = 3-4. *P < 0.05, 1-way ANOVA with Tukey's post hoc test. **(C-E)** Typical pictures exhibiting FOS (red) and TH (green) distribution in the VTA in the sham, resilient, and susceptible groups. Areas in the white rectangle are enlarged on the right. The double arrowheads indicate FOS/TH double-labeled neurons. The arrows indicate FOS single-labeled neurons. The arrowheads indicate TH single-labeled neurons. Scale bar: 100 μm (left), 20 μm (right). **(F** and **G)** Comparison of the number of FOS- and TH-positive neurons in the VTA. *n* = 3 mice (15 slices) per group. *P < 0.05, **P < 0.01, ****P < 0.0001, FOS: Kruskal-Wallis test with Dunn's post hoc test; TH: 1-way ANOVA with Tukey's post hoc test. **(H)** Ratio of FOS-positive cells in TH-positive and TH-negative neurons.

Based on the classification, we examined the expression level of TH and GAD67, markers for the two dominant neuronal types (DAergic and GABAergic neurons), in VTA in the sham, resilient, and susceptible groups. Western blotting revealed a significantly lower level of TH and higher expression of GAD67 in the susceptible group than in the sham or resilient group (Figure 3A-B). Double immunofluorescent staining for TH and FOS in the VTA showed that the numbers of TH-positive and FOS-positive neurons were also markedly decreased in the susceptible group compared with the sham or resilient group (Figure 3C-G). Further counting disclosed that the ratio of FOS-positive neurons showing TH immunoreactivity in the susceptible group was decreased to 20.2% ± 0.52%, compared with 32.5% ± 1.71% in the sham and 31.1% ± 4.11% in the resilient group, whereas the ratio of FOS-positive neurons showing TH-negative immunostaining was increased in the susceptible group (susceptible: 79.8% ± 0.52%; sham: 67.6% ± 1.71%; resilient: 69.0% ± 4.11%) (Figure 3H). Similarly, these changes, i.e., decreased numbers of TH- and FOS-positive neurons and lowered double-labeling rate in FOS-positive neurons in the susceptible group, were more prominently manifested in the mVTA than in the lVTA (Figure 3C-H and Figure S4). These results suggested that the activity of VTA DAergic neurons, especially those in the mVTA, undergoes differential plastic changes in neuropathic pain, i.e., being downregulated in the susceptible group but maintaining unchanged in the resilient group.

**Figure 4:**
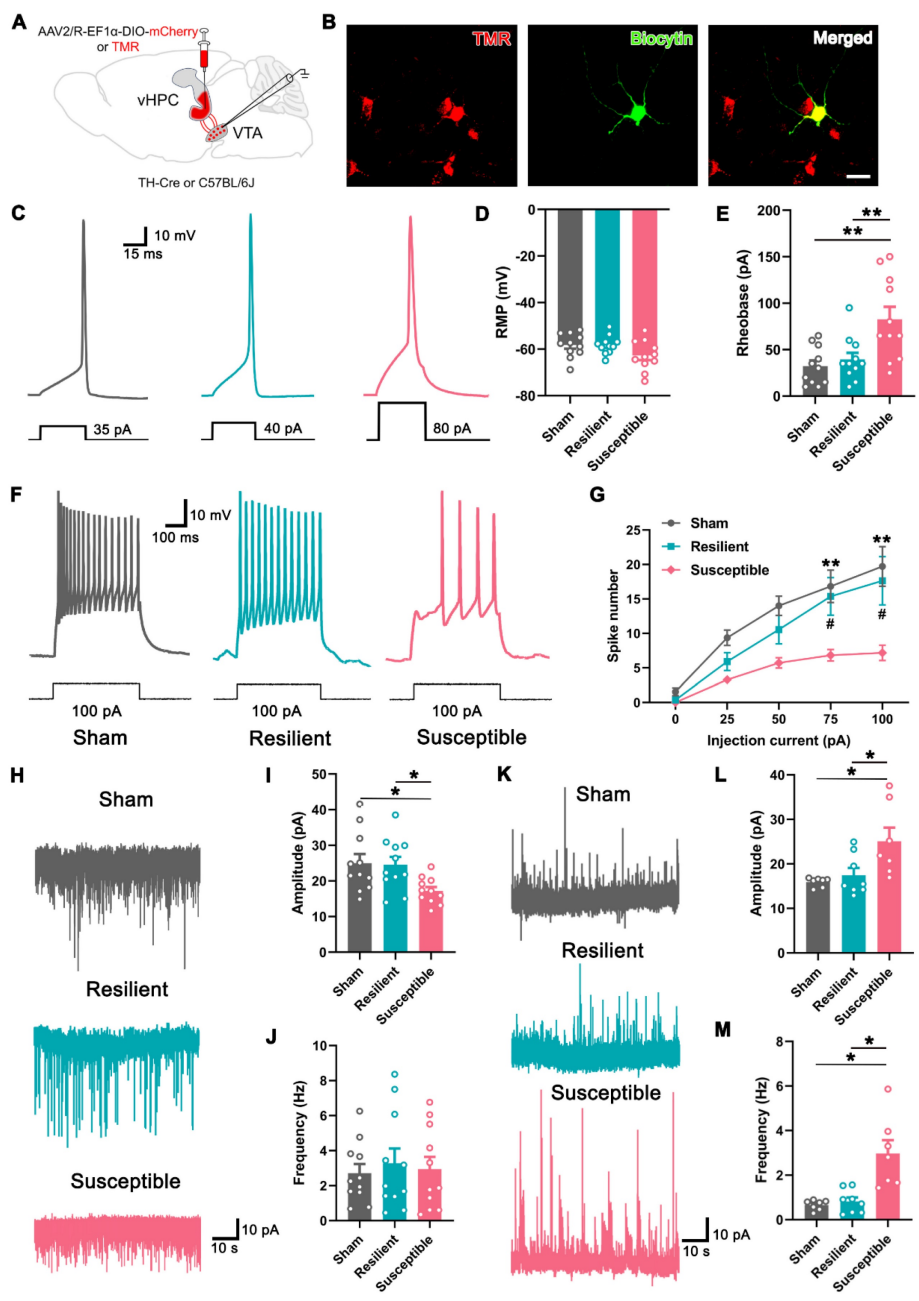
** Decreased excitability in VTA-vHPC projection neurons in susceptible mice. (A)** Schematic diagram showing the retrograde tracer (TMR) or AAV2/R-EF1α-DIO-mCherry injection in the vHPC of C57BL/6J or TH-Cre mice. The electrophysiological recording was performed by targeting the positive neurons in the VTA. **(B)** Typical pictures showing one recorded TMR (red)-labeled neuron infused with biocytin (green) in the VTA. Scale bar: 20 μm. **(C)** Representative traces of action potential induced by intracellular injection of depolarizing current (30 ms, 5 pA increments) in TMR^+^ neurons in the sham, resilient, and susceptible groups. **(D** and **E)** Summary of the resting membrane potential (RMP) and the rheobase of TMR^+^ neurons in three groups. *n* = 3 mice (11 TMR^+^ neurons) per group. **P < 0.01, by 1-way ANOVA with Tukey's post hoc test. **(F)** Typical firing activity of TMR^+^ neurons in response to 100 pA depolarizing currents in the sham, resilient, and susceptible groups. **(G)** Comparison of the spike number in response to step-wise depolarizing currents (400 ms, 0-100 pA) in TMR^+^ neurons of three groups. *n* = 3 mice (11 TMR^+^ neurons) per group. ^#^*P* <0.05, **P < 0.01 compared with the susceptible group, by 2-way RM ANOVA with Šidák's post hoc test.** (H)** Representative traces of sEPSC recorded in VTA-vHPC projection neurons (TMR^+^) from sham, resilient, and susceptible mice.** (I** and **J)** Summary of the amplitude and frequency of sEPSC in TMR^+^ neurons. *n* = 3 mice (11 TMR^+^ neurons) per group. *P < 0.05, by 1-way ANOVA with Tukey's post hoc test. **(K)** Representative traces of sIPSC recorded in VTA^DA^-vHPC projecting (mCherry-labeled) neurons from sham, resilient, and susceptible mice. **(L** and **M)** Summary of the amplitude and frequency of sIPSC in mCherry-labeled neurons. *n* = 3 mice (7-8 neurons) per group. *P < 0.05, by Kruskal-Wallis test with Dunn's post hoc test (L) and Welch's ANOVA test with Tamhane's T2 post hoc test (M).

We then examined the electrophysiological property of the vHPC-projecting VTA neurons in differentiated neuropathic pain. For this purpose, retrograde tracer tetramethyl rhodamine (TMR) was injected into the vHPC, and whole-cell patch clamp recording was performed seven days later (Figure 4A). As shown in Figure 4B, TMR^+^ neurons in slices containing the VTA could be detected under fluorescent microscopy, and the recorded neuron was verified by infusion with biocytin. First, we examined the intrinsic properties of TMR^+^ neurons in three groups by comparing the minimum current required to elicit an action potential (rheobase) in the current clamp mode. The results showed that the resting membrane potential of the labeled neurons was undifferentiated among the sham, resilient, and susceptible mice, but the rheobase of the projection neurons was markedly higher in the susceptible mice (Figure 4C-E). The spike numbers elicited by the injection of the depolarized step currents were also compared among the groups. As shown and summarized in Figures 4F and G, the susceptible mice exhibited significantly decreased spike numbers under 75 pA and 100 pA injection currents, compared with either sham or resilient group, indicating the lower responsivity of the VTA-vHPC projection neurons under depressive comorbidity.

Next, we recorded the spontaneous excitatory postsynaptic current (sEPSC) in the projection neurons in three groups in the voltage clamp mode. The results showed that the amplitude of sEPSC in the susceptible group was significantly decreased compared with the sham and resilient groups, indicating weakened excitatory postsynaptic responses (Figure 4H-I). By contrast, the frequency of sEPSC, which most likely represents the probability of presynaptic release of neurotransmitters, displayed no differences among the three groups (Figure 4J). We also examined the spontaneous inhibitory postsynaptic current (sIPSC) in vHPC-projecting DA neurons by injecting Cre-dependent AAV expressing mCherry (AAV2/R-EF1α-DIO-mCherry) in the vHPC of TH-Cre mice (Figure 4 A). As is evident from Figure 4K-M, the amplitude and frequency of sIPSC in the VTA^DA^ projection neurons were significantly higher in resilient mice than in the sham and susceptible groups. Together, these results strongly suggested that the activity of the vHPC-projecting VTA neurons is decreased in depressive comorbidity of neuropathic pain, which is at least partially mediated by the increased inhibitory drive from the intrinsic and/or extrinsic projections.

### Modulation of depression-like behaviors by the vHPC projection

**Figure 5:**
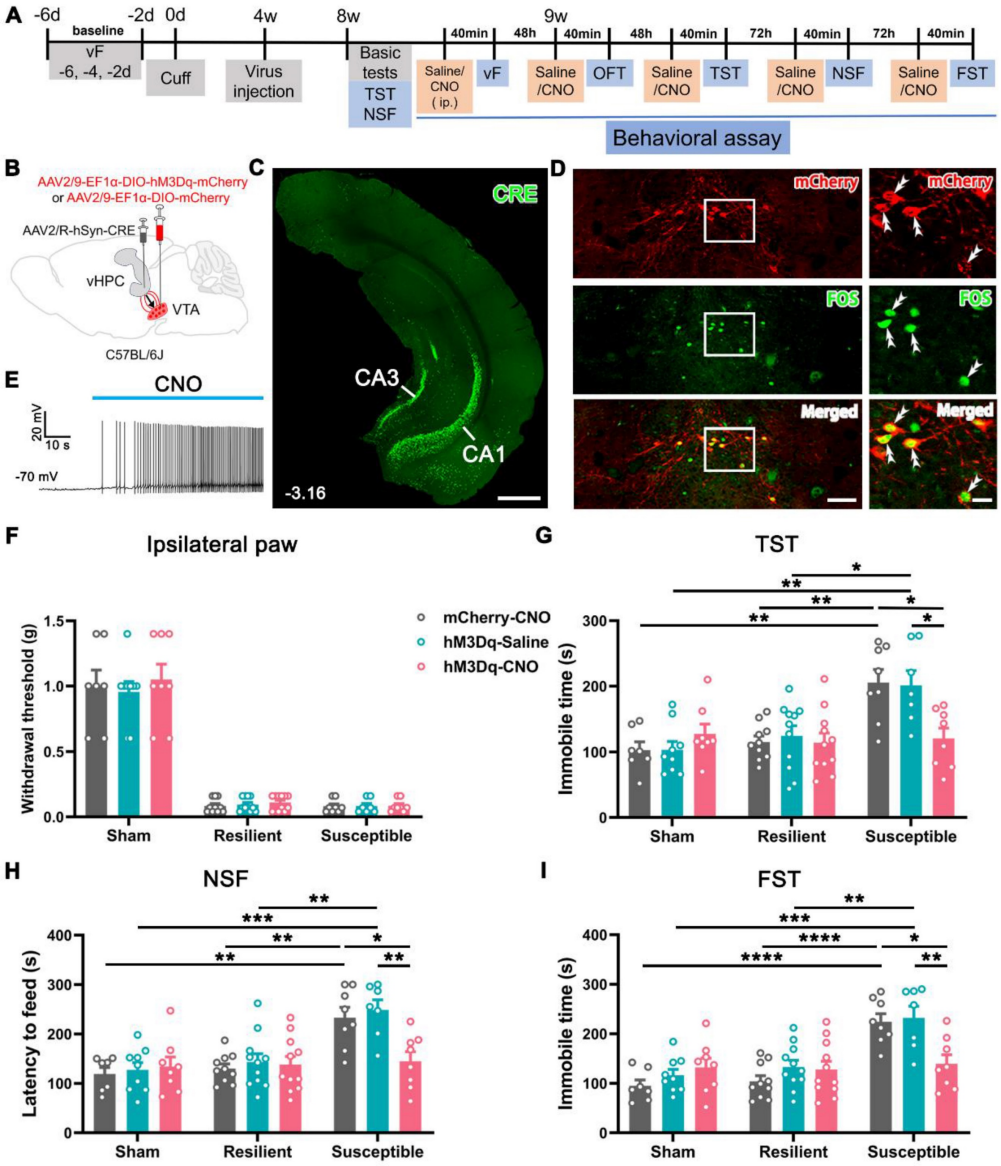
** Improvement in depression-like behaviors but not mechanical allodynia in susceptible mice by chemogenetic activation of VTA-vHPC projection neurons. (A)** Experimental design. **(B)** Schematic of virus injection. AAV2/R-hSyn-CRE was injected into the bilateral vHPC and AAV2/9-EF1α-DIO-hM3Dq-mCherry or AAV2/9-EF1α-DIO-mCherry was injected into the bilateral VTA. **(C)** Representative image showing the infection of AAV2/R-hSyn-CRE in the vHPC detected by CRE immunofluorescent staining (green). Scale bar: 500 μm. **(D)** Representative image of CRE-dependent expression of hM3Dq-mCherry (red) and CNO-induced FOS (green) expression in the VTA. The white rectangles are enlarged on the right, with double arrowheads indicating hM3Dq-mCherry/FOS double-labeled neurons. Scale bar: 200 μm (left), 50 μm (right). **(E)** Typical trace showing bath application of CNO (10 μM) enhanced neuronal discharge in hM3Dq-mCherry-expressing VTA neurons. Current clamp recording. **(F)** The effects of activating VTA-vHPC projection neurons on the mechanical threshold in sham, resilient, and susceptible groups. *n* = 7-11. **(G-I)** Activation of VTA-vHPC projection neurons ameliorated depression-like behaviors in the TST, NSF, and FST in the susceptible group. *n* = 7-11. *P <0.05, **P <0.01, ***P < 0.001, ****P < 0.0001, by 2-way ANOVA with Šidák's post hoc test.

Since the function of VTA^DA^ neurons and vHPC-projecting neurons was hyporegulated in the depressive comorbidity, we questioned whether activation of the pathway affects the neuropathic pain and the comorbid depression. To this end, cuff or sham mice at POW 4 were injected with AAV2/R-hSyn-CRE into the bilateral vHPC and AAV2/9-EF1α-DIO-hM3Dq-mCherry or the control virus (AAV2/9-EF1α-DIO-mCherry) into the bilateral VTA (Figure 5A-B). The injection site of Cre virus in the vHPC and Cre-dependent expression of hM3Dq-mCherry in the VTA are illustrated in Figures 5C and D. Application of clozapine N-oxide (CNO) induced FOS expression and increased spiking in hM3Dq-mCherry-expressing VTA neurons (Figure 5D-E), suggesting the success of the present chemogenetic system.

By a similar clustering method, the cuff mice were classified as the resilient and susceptible groups according to their performance in basic tests (TST and NSF) at POW 8 (Figure S5A-C). We examined the effects on pain threshold and mood-related behaviors after initiation of the DREADDs. The results showed that CNO injection (2 mg/kg) did not influence the pain sensitivity of the ipsilateral and contralateral hind paws in the sham, resilient, or susceptible mice compared with their respective control groups (Figure 5F and Figure S5D). The performance of animals in the total distance, central distance percentage, and central time in the OFT was also indiscriminate among different treatments in the sham, resilient, and susceptible groups (Figure S5E-G), indicating that activation of this pathway did not affect the locomotor activity and the anxious status of the mice. However, chemogenetic activation of vHPC-projecting VTA neurons in the susceptible mice (hM3Dq-CNO) significantly reduced the latency to feed in the NSF and the immobile time in the TST and FST compared with the saline-injected mice (hM3Dq-Saline) or the control virus group (mCherry-CNO) (Figure 5G-I). By contrast, the same treatment had no apparent effects in either the resilient or sham groups (Figure 5G-I). These results suggested that activation of vHPC-projecting VTA neurons specifically improves the depression phenotype in the susceptible group.

**Figure 6:**
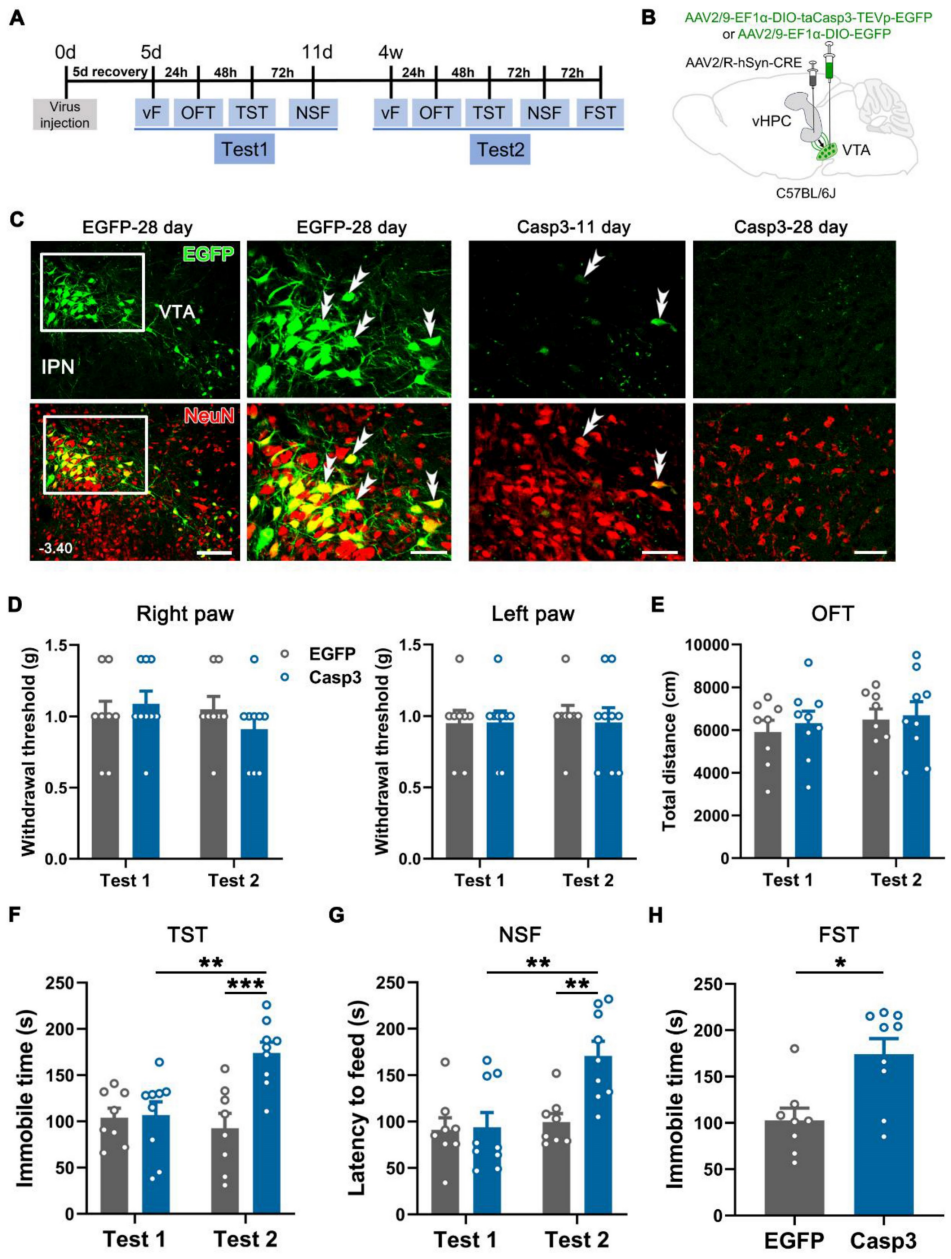
** Depression-like behaviors in naïve mice induced by Caspase 3-based ablation of VTA-vHPC projection neurons. (A)** Experimental design. **(B)** Schematic of virus-targeting in naïve mice. AAV2/R-hSyn-CRE was injected into the bilateral vHPC and AAV2/9-EF1α-DIO-taCasp3-TEVp-EGFP or AAV2/9-EF1α-DIO-EGFP into the bilateral VTA. **(C)** (Left) Representative image of CRE-dependent expression of control virus (green) and NeuN immunostaining (red) in the VTA on day 28 post-injection (EGFP-28 day). Scale bar: 100 μm. The white rectangular areas are enlarged on the right, with double arrowheads indicating EGFP/NeuN double-labeled neurons. Scale bar: 50 μm. (Right) The expression of taCasp3-TEVp-EGFP (green) and NeuN immunostaining (red) after 11 days (Casp3-11 day) or 28 days (Casp3-28 day) of virus injection. Note the compact alignment of neurons in the former and sparse neurons in the latter. Scale bar: 50 μm. **(D)** Effect of ablation on the mechanical threshold of right or left hind paws in Test 1 and Test 2. *n* = 8-9. 2-way RM ANOVA with Šidák's post hoc test. **(E)** The total distance in the OFT was similar between the EGFP and Casp3 groups in Test 1 and Test 2. *n* = 8-9. 2-way RM ANOVA with Šidák's post hoc test. **(F** and** G)** Apoptosis of VTA-vHPC projection neurons (Casp3-Test 2) induced increased immobile time in the TST and increased latency to feed in the NSF. *n* = 8-9. **P < 0.01, ***P <0.001, by 2-way RM ANOVA with Šidák's post hoc test. **(H)** Apoptosis of VTA-vHPC projection neurons induced increased immobile time in the FST compared with the control virus group. *n* = 8-9. *P < 0.05, by Mann-Whitney test.

Next, we examined whether the VTA-vHPC projection was obligatory to maintain normal emotion by selectively ablating vHPC-projecting VTA neurons in naïve mice (Figure 6A). AAV2/R-hSyn-CRE was bilaterally injected into the vHPC, and AAV2/9-EF1α-DIO-taCasp3-TEVp-EGFP (Casp3 group) or the control virus (AAV2/9-EF1α-DIO-EGFP) was bilaterally delivered into the VTA (Figure 6B). Immunofluorescent staining for NeuN was performed to verify whether the Caspase 3-based strategy could induce apoptosis. As illustrated in Figure 6C, many control virus-infected cells were observed in the mVTA on day 28 (Test 2 started), which showed an intact and elegant appearance and were NeuN-positive. By contrast, clear Casp3-EGFP and NeuN double-labeled neurons in the mVTA were barely visible on day 28 (Figure 6C, right panel). Instead, a relatively sparse alignment of neurons was observed in the Casp3 group, suggesting the loss of partial VTA neurons. Interestingly, a few double-labeled neurons were observed in the Casp3 group on day 11 (Test 1 finished), indicating sporadic infection by the virus without influencing the integrity of the whole neuronal population. These results demonstrated that Test 1 (during 5-11 days) was based on the intact function of VTA-vHPC projection, while Test 2 (after 28 days) was after the ablation of the neurons.

The von Frey filament test showed that ablation of the projection neurons on day 28 did not affects the mechanical sensitivity in the bilateral hind paws of the infected mice compared with the control group or the Casp3 mice in Test 1 (Figure 6D). Likewise, the ablating treatment did not affect the locomotor activity and anxious status of the animals as evaluated in the OFT (Figure 6E and Figure S6A-B). However, we observed significantly enhanced immobile time in the TST and increased latency to feed in the NSF in the Casp3 group compared with the control group or the Casp3 group in Test 1 (Figure 6F-G). The increased immobile time was also observed in the FST in the Casp3 group compared with the control group (Figure 6H). Taken together, the loss-of-function modulation suggested a pivotal role of VTA-vHPC projection in maintaining the mood balance of naïve mice.

### Reversal of comorbid depressions by phasic activation of VTA^DA^-vHPC projection

**Figure 7:**
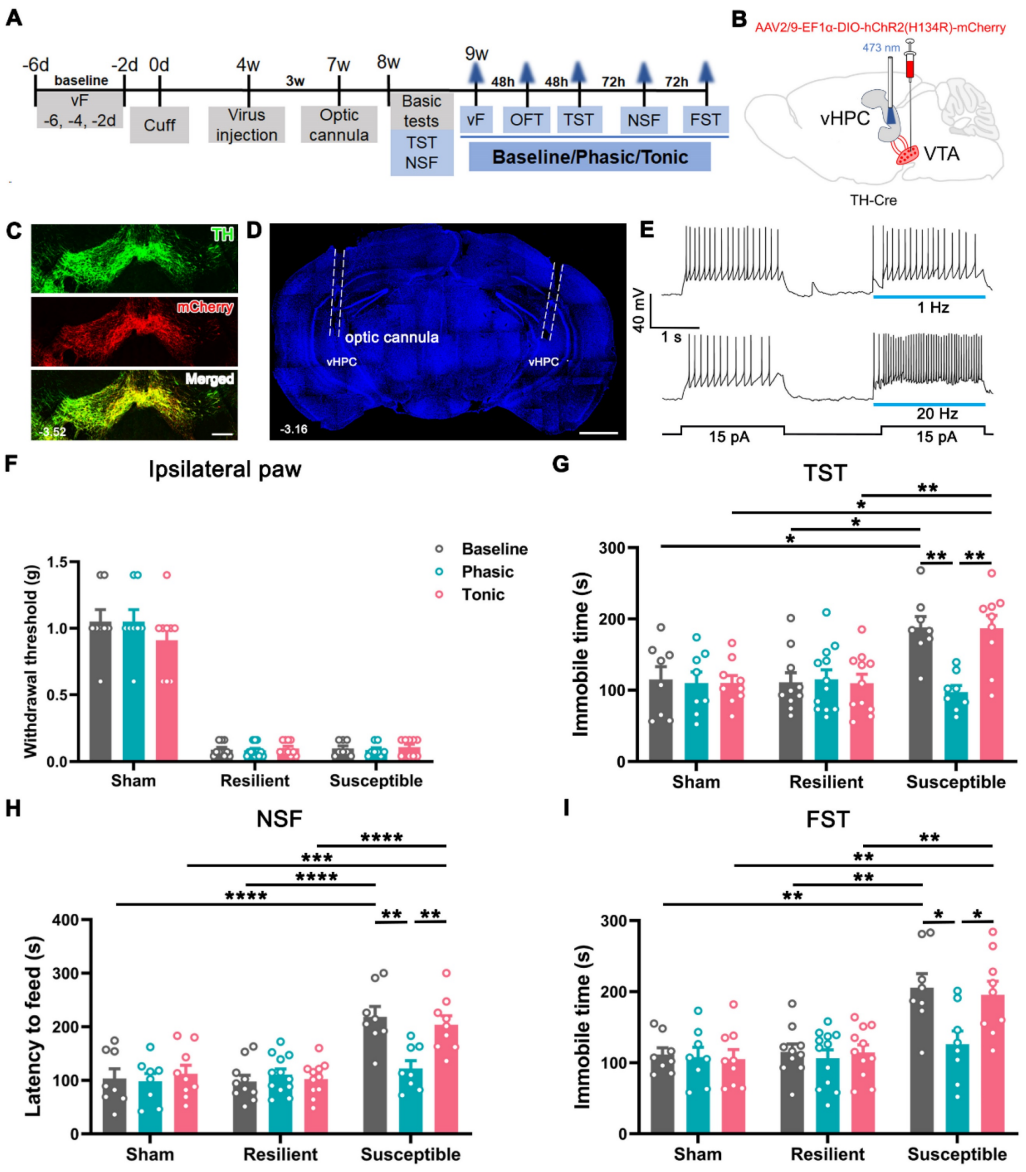
** Amelioration of depression-like behaviors but not mechanical allodynia by optogenetic activation of the VTA^DA^-vHPC pathway in susceptible mice. (A)** Experimental schedule. **(B)** Schematic of experimental strategy. AAV2/9-EF1α-DIO-hChR2(H134R)-mCherry was injected into the bilateral VTA, and optic cannulae were implanted into the bilateral vHPC in TH-Cre mice. **(C)** Typical picture showing ChR2-mCherry expression in the VTA. Most infected neurons were TH immunoreactive. Scale bar: 200 μm. **(D)** Representative image showing the implantation site of the optic cannula in the vHPC. Scale bar: 1 mm.** (E)** Sample traces of the evoked action potentials induced by 15 pA depolarized current without or with blue light (473 nm, 2 s, blue bars) at 1 Hz or 20 Hz. The laser was delivered 100 ms ahead of the current injection.** (F)** Laser stimulation of VTA^DA^-vHPC pathway failed to affect the mechanical threshold in the ipsilateral hind paw in nine groups. *n* = 8-12. **(G-I)** Comparison of the effect of laser stimulation on cuff-induced depressive comorbidity. Phasic but not tonic laser stimulation significantly decreased the immobile time in the TST and FST and the latency to feed in the NSF in the susceptible group. *n* = 8-12. *P < 0.05, **P < 0.01, ***P <0.001, ****P < 0.0001, by 2-way ANOVA with Šidák's post hoc test.

As 91.7% of vHPC-projecting VTA neurons are DAergic, we investigated whether the anti-depressant function of the VTA-vHPC pathway involves the contribution of GABAergic and glutamatergic neurons by optogenetics in transgenic mice. Cre-dependent AAV expressing channelrhodopsin-2-mCherry (AAV2/9-EF1α-DIO-hChR2(H134R)-mCherry) was injected into the bilateral VTA in the TH-Cre mice (Figure 7A-C). Optic cannulae were implanted over the vHPC one week before behavioral assessments to deliver phasic (20 Hz) or tonic (1 Hz) laser stimuli at 473 nm (Figure 7D). We first tested the specificity and effectiveness of the strategy. Immunofluorescent staining showed that most ChR2-mCherry-incorporated neurons were TH-positive, suggesting specific expression of ChR2 in DAergic neurons (Figure 7C). Patch clamp recording revealed that spiking induced by injecting the depolarized current was potentiated by the application of blue light at 20 Hz other than 1 Hz, indicating the efficacy of the 20 Hz blue laser stimuli in exciting the ChR2-expressing neurons (Figure 7E). Similarly, the cuff mice were distinguished into the resilient and susceptible groups according to the K-means analysis of animal performance features from basic tests at POW 8 (Figure S7A-C). Every group, including the sham, was further divided into 3 subgroups based on the treatment applied, i.e., baseline (no-light), phasic (phasic laser stimulation), and tonic (tonic laser stimulation) (Figure 7F-I).

We examined the influence of photostimulation on pain, anxiety- and depression-like behaviors since POW 9. As shown in Figure 7F and Figure S7D, neither phasic nor tonic stimulation affected the mechanical threshold in both ipsilateral and contralateral hind paws in the sham, resilient, and susceptible mice. Likewise, both treatments failed to alter the animal performance of all groups in the OFT (Figure S7E-G). In contrast, phasic stimulation significantly decreased the latency to feed in the NSF and the immobile time in the TST and FST in the susceptible mice compared with the baseline group (Figure 7G-I). However, tonic stimulation caused no observable effects on the animal performance of the susceptible mice in the NSF, TST, and FST (Figure 7G-I). Notably, the behavioral manifestations of the sham and resilient mice in the NSF, TST, and FST were not significantly different among the baseline, phasic, and tonic treatments. These optogenetic results revealed that phasic stimulation of VTA^DA^-vHPC projection efficiently improved depression but not pain or anxiety in cuff mice.

### Involvement of D1 and D2 receptors in the anti-depressive function of VTA^DA^-vHPC projection

**Figure 8:**
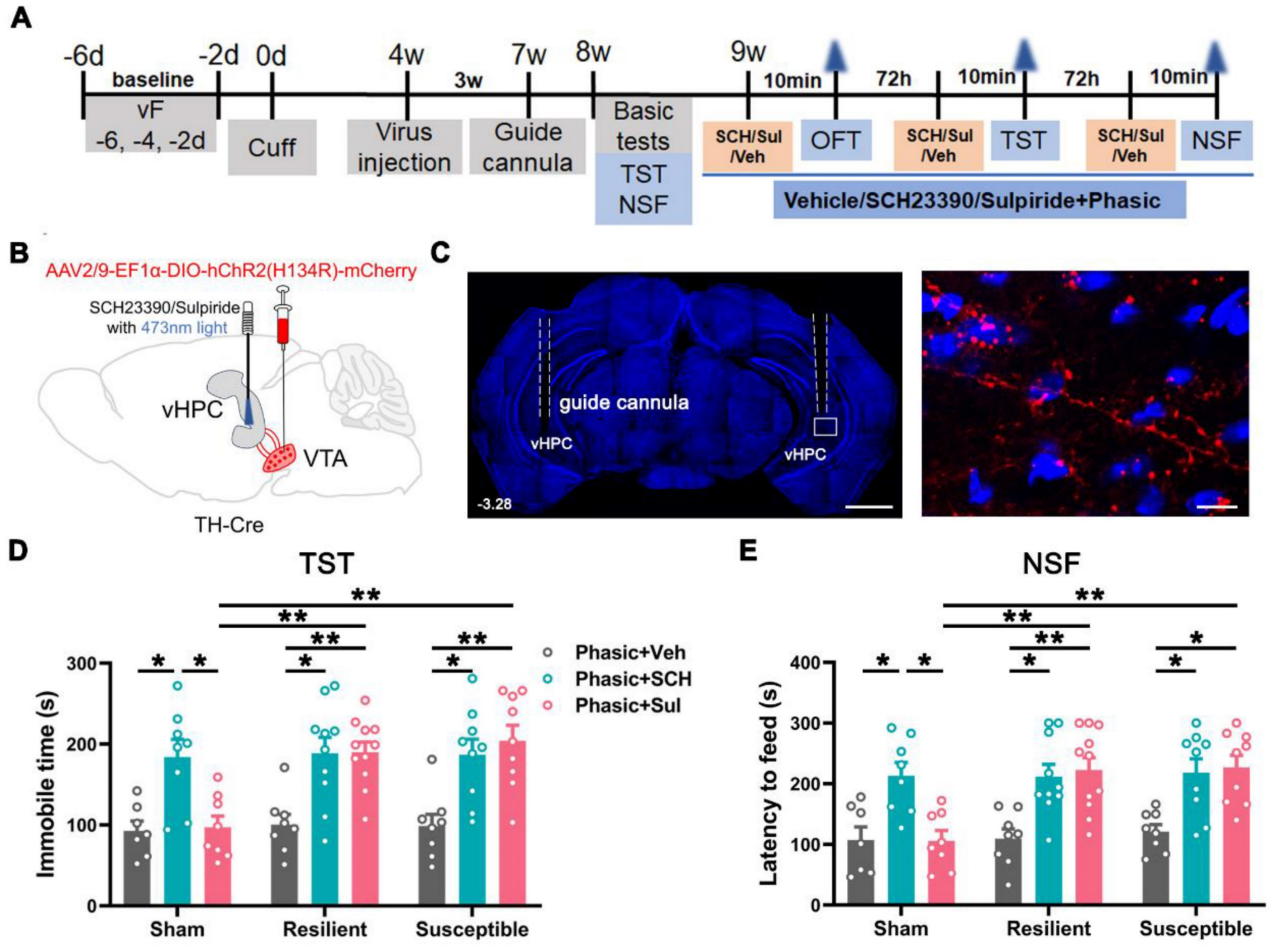
** Effect of phasic activation of the VTA^DA^-vHPC pathway via D1 or D2 receptors. (A)** Experimental design. **(B)** Schematic of virus injection and laser and drug delivery. AAV2/9-EF1α-DIO-hChR2(H134R)-mCherry was injected into the bilateral VTA, and guide cannulae were implanted into the bilateral vHPC in TH-Cre mice. **(C) (**Left) Representative image showing the implantation site of guide cannulae in the vHPC. Scale bar: 1 mm. (Right) Amplified image shown by the white rectangle on the left. ChR2-mCherry labeled fibers and terminals formed clusters around DAPI-stained nuclei in the vHPC. Scale bar: 50 μm. **(D** and **E)** Effect of application of either D1R or D2R antagonist on animal behaviors in different groups. Delivery of SCH23390 or sulpiride blocked the anti-depression effect of phasic stimulation in the susceptible group. Drug delivery also caused depression-like behaviors in the resilient and sham groups. *n* = 7-11. *P < 0.05, **P < 0.01, by 2-way ANOVA with Šidák's post hoc test.

Dopamine 1 and 2 receptors (D1R and D2R) play pivotal roles in modulating neuronal excitability and synaptic plasticity in the hippocampus 18, 24, 25. We investigated the receptors involved in the anti-depressive function of VTA^DA^-vHPC projection by combining optogenetics with behavioral pharmacology. The experimental protocol used was similar to the above optogenetics (Figure 8A-B). The position of the guide cannula was confirmed at the end of all behavioral tests, and the ChR2-mCherry-labeled projecting fibers and terminals were found to cluster around DAPI-stained nuclei in the vHPC (Figure 8C).

Following basic tests at POW 8 for animal grouping (Figure S8A-C), 0.1 μg D1R antagonist SCH23390 (SCH, 0.05 μg/hemisphere, 0.3 μL), 0.2 μg D2R antagonist sulpiride (Sul, 0.1 μg/hemisphere, 0.3 μL), or vehicle (Veh, saline) was microinjected 10 min before laser stimulation into the vHPC of the sham, resilient, and susceptible mice, respectively (Figure 8A-B). As expected, the combined treatments had no discernible influences on the locomotor activities and anxious status of the sham, resilient, and susceptible mice in the OFT (Figure S8D-F). However, pre-application of SCH23390 or sulpiride effectively and equally antagonized the antidepressant effect produced by the phasic stimulation of the VTA^DA^-vHPC pathway in the susceptible mice (Figure 8D-E). Surprisingly, the application of the two antagonists also induced depression-like behaviors in the resilient mice, suggesting the essential involvement of the two receptors in the maintenance of animal resilience to depressing stress. It is worth noting that the application of SCH23390 but not sulpiride also induced a depressive phenotype in the sham mice, indicating the importance of D1R but not D2R in mood regulation under normal conditions (Figure 8D-E). These data suggested that D1 and D2 receptors equally contribute to the anti-depressive effect produced by phasic stimulation of the VTA^DA^-vHPC projection.

### The low-segregated D1R in pyramidal neurons and highly-segregated D2R in GABAergic neurons

To explore the possible mechanisms underlying behavioral pharmacology, we examined D1R and D2R expression in the vHPC by fluorescence *in situ* hybridization (FISH) combined with immunofluorescent staining. The specificity of FISH was verified in the NAc, an important striatal region containing high levels of D1R and D2R, using D1R-tdTomato and D2R-EGFP mice. As shown in Figure S9, these two mouse lines contained high immunostaining signals of tdTomato and EGFP in the NAc, signifying the reliable expression of D1R and D2R. FISH with the D1R probe resulted in 84.42% of double-labeling signals in D1R-tdTomato mice, and FISH with the D2R probe caused 86.67% of double-labeling in D2R-EGFP mice, suggesting the specificity of the used probes.

**Figure 9:**
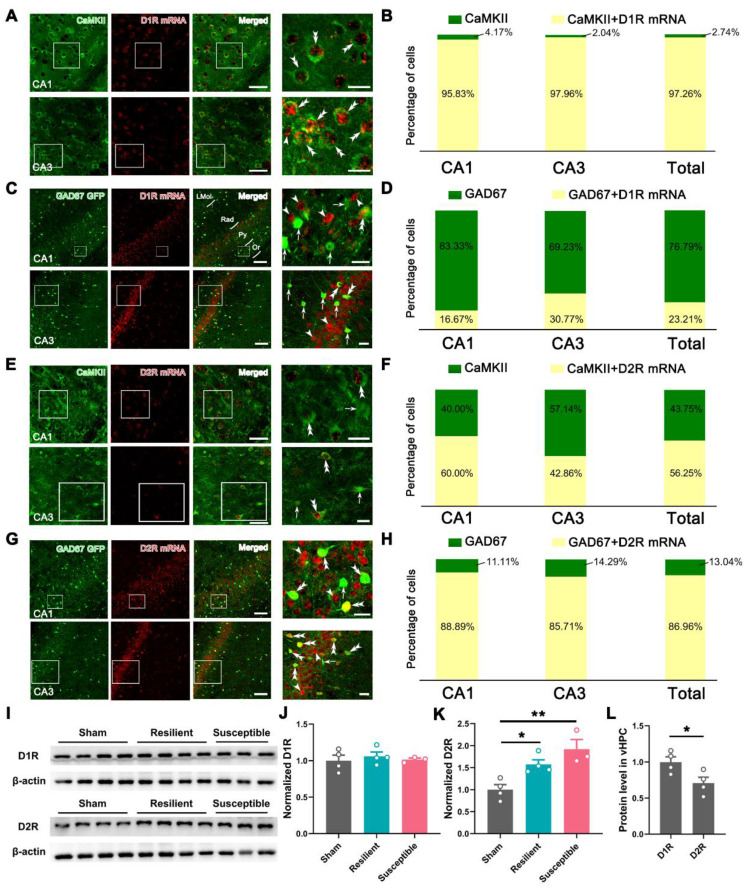
** Expression pattern of D1R and D2R in pyramidal and GABAergic neurons of vHPC. (A** and** C)** Typical images exhibiting the distribution of CaMKII immunoreactivities (green), GFP immunoreactivities (green), and D1R mRNA signals (red) in the CA1 and CA3 of vHPC. Areas in the white rectangle are enlarged on the right. The double arrowheads indicate CaMKII/D1R or GFP/D1R double-labeled neurons. The arrowheads indicate D1R single-labeled neurons. The arrows indicate GFP single-labeled neurons. **(B** and **D)** Percentage of CaMKII or GAD67-GFP single-labeled and CaMKII/D1R or GAD67-GFP/D1R double-labeled neurons. **(E** and **G)** Representative images showing the distribution of CaMKII immunoreactivities (green), GFP immunoreactivities (green), and D2R mRNA signals (red) in the CA1 and CA3 of vHPC. Areas in the white rectangle are enlarged on the right. Double arrowheads indicate CaMKII/D2R or GFP/D2R double-labeled neurons. Arrows indicate CaMKII or GFP single-labeled neurons. Arrowheads indicate D2R single-labeled neurons. Scale bar (A and E): 50 μm (left), 20 μm (right); Scale bar (C and G): 100 μm (left), 20 μm (right). **(F** and **H)** Percentage of CaMKII or GAD67-GFP single-labeled and CaMKII/D2R or GAD67-GFP/D2R double-labeled neurons. **(I)** Typical band for Western blotting of D1R and D2R levels in vHPC. **(J** and** K)** Comparison of the relative levels of D1R and D2R in the three groups. *n* = 3-4. *P < 0.05, **P < 0.01, 1-way ANOVA with Tukey's post hoc test.** (L)** Comparison of D1R and D2R in the sham group. *n* = 4. *P < 0.05, unpaired Student's *t*-test.

Further, FISH of D1R with immunostaining of Ca^2+^/calmodulin-dependent kinase II (CaMKII), a marker of pyramidal neurons in the cortex, generated a hybridization signal in both CA1 and CA3, with the averaged double-labeling rate reaching 97.26% in CaMKII-positive neurons (Figure 9A-B). By contrast, FISH of D1R in GAD67-GFP mice only caused an average of 23.21% of hybridization signal in GFP-labeled GABAergic neurons (Figure 9C-D), suggesting a dominant or preferential location of D1R in pyramidal neurons of the vHPC. A similar protocol targeting D2R with CaMKII immunostaining showed a substantial but lower double-labeling rate in the pyramidal neurons (56.25%) (Figure 9E-F). In contrast, FISH in GAD67-GFP mice resulted in 86.96% of D2R detection in GABAergic neurons (Figure 9G-H). These results suggested a low-segregated expression of D1R in pyramidal neurons and a highly-segregated expression of D2R in GABAergic neurons.

We finally assessed the dynamic change of D1R and D2R in the sham, resilient, and susceptible groups by Western blotting. As illustrated in Figure 9I-K, the D1R level showed subtle alterations among the three groups, while the expression of the D2R underwent significant enhancement in the two model groups. We also compared the level of D1R and D2R in the sham group and found a relatively decreased expression of the latter in the vHPC (Figure 9L).

**Figure 10:**
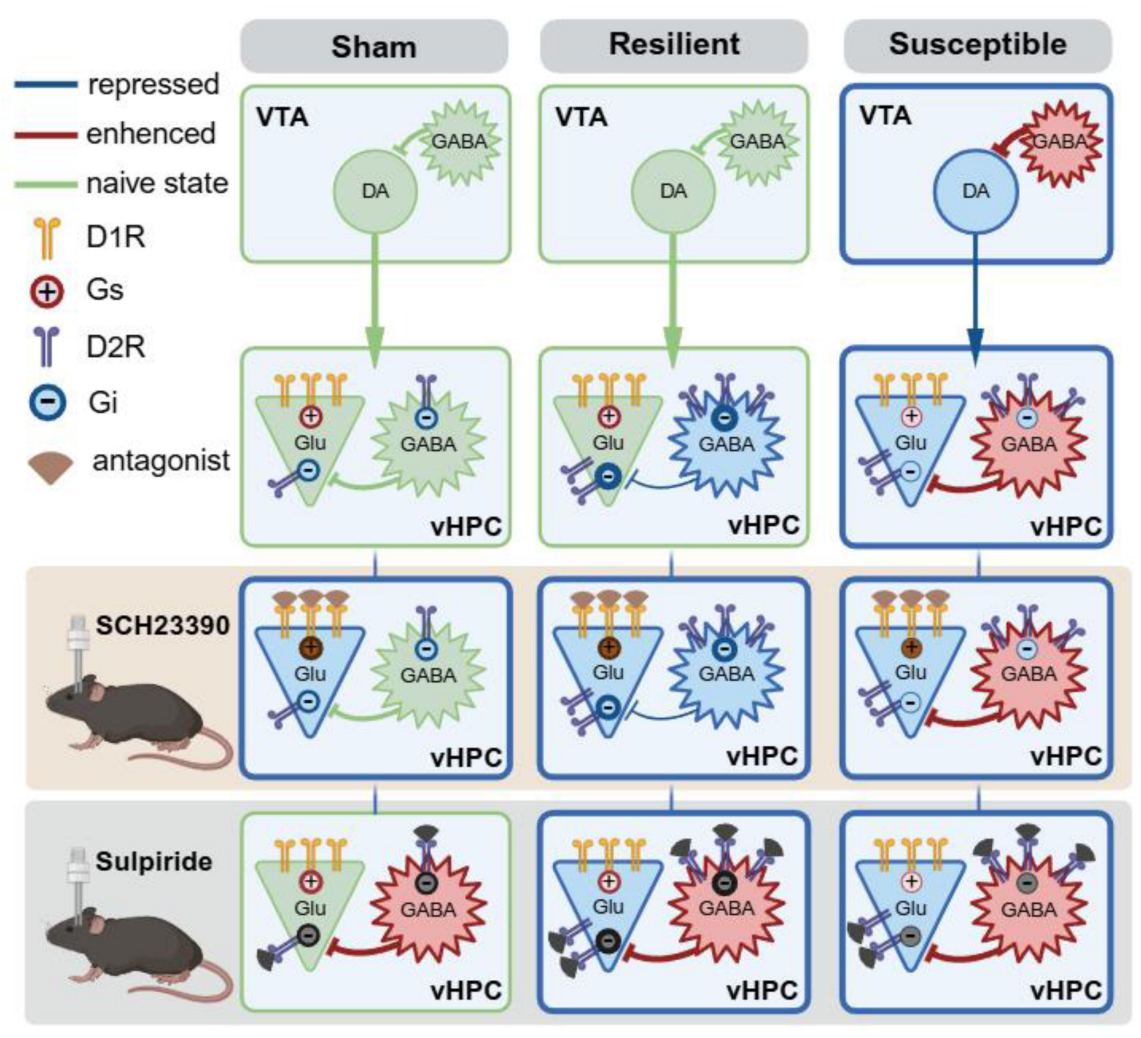
** Working hypothesis for the anatomical feature and adaptive changes of the VTA^DA^-vHPC pathway in differentiated neuropathic pain.** Receptor mechanisms underlying the boosting of VTA^DA^-vHPC tone in the sham, resilient, and susceptible mice are also illustrated. Created in https://BioRender.com.

Based on the cell-specific detection of D1R and D2R in naïve mice and the dynamic changes of the two receptors following cuff, we proposed a working hypothesis to explain the circuitry and receptor mechanisms underlying the anti-depressive effect of boosting VTA^DA^-vHPC projection (Figure 10, see discussion for details).

## Discussion

Dysfunction of the mesolimbic system contributes to neuropsychiatric diseases, such as major depressive disorder and addiction 4. Emerging evidence has pointed to an essential role of the system in manipulating chronic pain symptoms, with emphasis on the VTA-NAc, PFC, ACC, and amygdala projections 4, 26. Our present study shows, for the first time, that the disrupted VTA^DA^-vHPC projection contributes to comorbid depression but not pain induced by a neuropathic 'cuff' model.

### A medially dominant VTA^DA^ projection to the vHPC

One essential feature of the pathway from VTA to the vHPC is the medially dominant location of the projection neurons. VTA is the brain region with high heterogeneity 6. To elucidate the connectivity of NAc with VTA subpopulations, Yang *et al.* divided VTA into mVTA and lVTA, with mVTA mainly encompassing the paranigral nucleus and interfascicular nucleus, and lVTA the lateral parabrachial pigmented nucleus and the medial lemniscus adjacent to the substantia nigra (SN) 21. They showed that neurons in NAcMed preferentially exert direct inhibitory control over mVTA DAergic neurons. In contrast, those in the lateral shell of NAc (NAcLat) mainly synapse onto lVTA GABAergic neurons. Moreover, a study demonstrated that the mVTA^DA^-NAcMed and lVTA^DA^-NAcLat pathways encode stress relief to prevent anhedonia and despair, respectively 20.

Based on this cellular and circuitry distinction, we first mapped the distribution of retrogradely labeled neurons in the VTA and found that 79% of FG-containing neurons were medially located. Interestingly, the projection neurons in the rostral VTA were mainly localized in the lateral part of mVTA, whereas those in the middle and caudal VTA were more prominent in the medial part of mVTA. Mesencephalic dopamine neurons are composed of the A8 region in the retrorubral field, the A9 region in the SN, and the A10 region in the VTA 15. Previous studies reported that the dopaminergic afferents to the dHPC mainly originated from rostral parts of A10 and A9 regions 15, 27, 28. The mediolateral organization of vHPC projection neurons was somehow similar to that of dHPC, as neurons in the lateral part of mVTA were near to or even partially mixed with SN in the rostral planes (Figure S1A-B). Nevertheless, we also observed a substantial number of medially located DA projection neurons in the middle and caudal VTA, reflecting the differential topology of VTA^DA^ projection along the dorsoventral axis of the HPC.

The second feature of the VTA-vHPC pathway is the DAergic dominant projection. Traditionally, DAergic neurons are proposed to constitute the main output of the VTA. Increasing evidence, however, suggests that glutamate and GABA neurons send long-range projections to many brain structures 6. By double immunofluorescent staining for FG and TH in GAD67-GFP knockin mice, we clarified that over 90% of vHPC-projecting neurons were DAergic. These results were consistent with a previous study reporting that partial VTA-vHPC projection neurons were TH-positive 29. By anterograde AAV tracing in TH-Cre mice, we further demonstrated that VTA^DA^ fibers or terminals were prominently distributed in the pyramidal layers of CA1 and CA3 regions and formed close contacts with neurons therein and with neurons in other strata (Figure 1G). In addition to numerous pyramidal neurons assembling neatly in the stratum pyramidale, the hippocampus also incorporates various populations of GABAergic interneurons, which mostly scatter in other strata such as oriens, rediatum, and lacunosum-moleculare 30. Our results thus suggested that the VTA^DA^ terminals may target pyramidal and GABAergic neurons in the vHPC.

### Hypoactive VTA^DA^-vHPC projection in comorbid depression

The reason why nearly half of the patients with chronic pain develop comorbid depression and others do not, remains unclear. A vulnerability or diathesis that puts an individual at increased risk of depression may be one possibility 5. To address this, we divided the cuff mice into the resilient (mice without depression-like behaviors) and susceptible (mice with depression-like behaviors) groups using the K-means algorithm. About 40% of mice with neuropathic pain displayed stable and long-lasting depression-like behaviors. The incidence of depression was comparable to the clinical reports 5, suggesting the validity of the model in manifesting mood disturbance comorbid with chronic pain.

Western blotting and immunofluorescent histochemical staining demonstrated the overall hypoactivity of the VTA^DA^ neurons in the susceptible mice, evidenced by the decrease in the TH expression level, the number of TH-positive neurons, and the ratio of TH/FOS double-labeled neurons. Further, the VTA-vHPC projection neurons exhibited increased rheobase value, lower spiking potency to injected current, and decreased amplitude of sEPSC in the susceptible group by patch clamp recording, potently demonstrating a hypodopaminergic connectivity from VTA to vHPC under comorbid depression. VTA^DA^ neurons receive abundant GABAergic inputs from brain regions such as the rostromedial mesopontine tegmental nucleus, periaqueductal grey, dorsal raphe nucleus, lateral hypothalamus, and ventral pallidum 6. In addition, axon terminals from local GABAergic neurons establish symmetric synapses and exert inhibitory actions on the dendrites of VTA^DA^ neurons through GABA_A_ receptors 6, 31, 32. Our group observed enhanced activity of VTA GABAergic neurons in comorbid depression and demonstrated that this excitation was mediated by increased glutamatergic inputs from the lateral habenula nucleus 33. Consistent with these observations, the current study showed that the expression level of GAD67, an enzyme isoform for GABA biosynthesis, was significantly elevated in the VTA in comorbid depression. The frequency and amplitude of sIPSC recorded from the VTA^DA^-vHPC projection neurons were also significantly increased in the susceptible mice. These results substantiated that the low activity of DAergic neurons is at least partially attributed to the allostatic hyperactivity of local or extrinsic GABAergic neurons.

In contrast to the susceptible mice, the activity of VTA^DA^ neurons and vHPC-projecting neurons in the resilient mice remained relatively intact, manifested by the similarities with the sham mice in TH expression, number of TH-positive neurons, the ratio of TH/FOS double-labeled neurons, level of GAD67, and excitability of the projection neurons. Hypodopaminergic conditions in chronic pain have been reported in preclinical and clinical studies 34, 35. For example, patients with fibromyalgia showed reduced activity in the VTA and decreased presynaptic dopamine metabolism in several brain regions, including the hippocampus 36, 37. Similarly decreased presynaptic dopamine function in the right putamen was demonstrated in patients with burning mouth syndrome 38. Our results seem to be at odds with these previous reports. However, careful comparison reveals that many of these studies did not distinguish the pathophysiology of the DA system according to the phenotype difference, i.e., pain with or without mood disturbance. Given the frequent occurrence of comorbid anxiety and/or depression in chronic pain, it is likely that the dysregulated DA status in these studies may involve the influence of mood alteration. In support of this, Hipólito *et al.* 39 found that the basic level of DA in the NAc in the CFA-induced inflammatory pain at two days post-injection was indifferent from that in control, suggesting the relatively intact resting VTA^DA^-NAc function in the early pain stage. Tiemann *et al.* 40 demonstrated that dopamine precursor depletion in healthy human volunteers influences pain affect but not pain sensation.

Together, the present results demonstrate a disrupted function of vHPC-projecting VTA^DA^ neurons in the susceptible but not resilient mice, thus extending our knowledge of the plastic changes of the DA system in response to chronic pain with or without affective disturbance.

### Improvement of comorbid depression by activation of the VTA^DA^-vHPC pathway

Since susceptible animals showed maladaptive changes in VTA^DA^-vHPC projection, we modulated the VTA-vHPC pathway and assessed the behavioral influences by three strategies. The chemogenetic and optogenetic experiments showed that activation of VTA-vHPC projection was sufficient to reverse depressive comorbidity without discernible influences on pain sensitivity. Caspase 3-based ablation of VTA-vHPC projection neurons successfully induced depression-like but not pain behaviors in naïve mice. These results demonstrated that the hypoactivity of the VTA^DA^-vHPC pathway plays a determinant role in the generation of depression-like phenotype other than sensory pain.

NAc and mPFC are two major components of the mesolimbic circuit. It was reported that high-frequency stimulation of VTA, which has been shown to increase DA concentrations in the PFC, significantly inhibited the nociceptive response in the PFC induced by acute noxious stimulation via D2R activity 41. Phasic activation of the VTA^DA^-mPFC pathway alleviated spared nerve injury-induced mechanical hypersensitivity by enhancing the activity of mPFC pyramidal neurons projecting to the ventrolateral periaqueductal gray (vlPAG), a key brain region involved in descending pain modulation 42. Interestingly, activation of glutamatergic projection from VTA to mPFC also alleviated spared nerve injury-induced neuropathic pain, possibly through restoring the mPFC outputs to the ACC 43. Besides the sensory modulatory role of the VTA-NAc pathway illustrated in the Introduction, these reports highlight the common role of the two VTA pathways in regulating the sensory component of pain in addition to any possible cognitive and affective influences.

By contrast, manipulation of the VTA^DA^-vHPC pathway solely influenced the comorbid depression without affecting the sensory pain in the present study. The functional preference of varied projections might be related to the structural similarity or isolation of the pathways. By retrograde triple labeling, we observed that 60% of mPFC-projecting VTA neurons sent collaterals to the NAc, which accounted for about 6% of the NAc-projecting VTA neuronal population. In contrast, few vHPC-destination neurons sent collaterals to either mPFC or NAc. Therefore, the VTA-vHPC pathway seems to emanate from a rather 'independent' cellular population, with few overlaps with the two mesolimbic projections. Presumably, VTA^DA^ neurons fine-tune adaptive behaviors through such shunting mechanisms by recognizing the sensation and affection components of chronic pain.

### Parallel involvement of D1R and D2R in the anti-depressant effect

DA signals through binding to five subtypes of metabotropic DA receptors, D1-D5, among which the brain mainly contains excitatory D1 and inhibitory D2 receptors 44. Numerous studies have elucidated the expression pattern and cellular location of D1R and D2R in the dHPC by autoradiography, in situ hybridization, immunohistochemistry, and, more recently, by genetic labeling 45, 46. Comparatively, the expression profile of D1 and D2 receptors in the vHPC is less concentrated than in the dHPC. Using transgenic mice, Wei *et al.* 47 showed that D1R and D2R were highly expressed in the pyramidal neurons of ventral CA1 and subiculum areas. By FISH and immunofluorescent staining in naïve mice, we confirmed D1R and D2R expression in pyramidal neurons. Moreover, in contrast with the highly-segregated expression of D1R and D2R in the striatum 48, our results disclosed that partial vHPC pyramidal neurons co-express both D1R and D2R, although the level of the latter was lower than the former. The low-segregated expression pattern of dopamine receptors was also reported in the dorsal CA1, where a high fraction of D1R-containing GABAergic neurons also expressed D2R 46.

In addition to pyramidal neurons, we detected more dominant D2R than D1R signals in GABAergic neurons, indicating a highly-segregated D2R expression in the inhibitory neurons. Following cuffing, D1R underwent minor alterations, while the level of D2R was significantly increased in the two cuff groups. Similarly, patients with atypical facial pain exhibited increased D2R availability in the left putamen 49. In conjunction with the close contacts formed between VTA^DA^ terminals and pyramidal neurons and interneurons, the results collectively suggested that a complex postsynaptic mechanism on excitatory and inhibitory neurons may also contribute to the adaptation of the VTA^DA^-vHPC pathway in neuropathic pain.

D1R and D2R play important roles in modulating synaptic plasticity in the hippocampus. Earlier evidence proved that D1-type receptors (including D1R and D5R) facilitated long-term potentiation (LTP) in the CA1 via protein kinase A activation, thereby retaining long-term memories 50. A recent study further revealed that photostimulation of VTA^DA^ terminals in the dHPC effectively triggered LTP at coactivated Schaffer collaterals (CA3-CA1 input) in a D1R-dependent manner, thus demonstrating a modulatory role of VTA-derived DA signaling in the contextual learning 51. In addition to D1R, systemic administration of the D2R/D3R agonist quinpirole has been proven effective in reversing nerve injury-caused disruption in hippocampal spike activity and working memory 17. These observations implied a coincident role of D1R and D2R signaling in improving synaptic transmission and hippocampal function.

Combining intrahippocampal injection of D1R or D2R antagonist with optogenetics, we demonstrated that pharmacologically antagonizing either D1R or D2R blocked the anti-depressive effects induced by phasic stimulation of the VTA^DA^-vHPC pathway in the susceptible group, suggesting a similar and parallel involvement of the two receptors in boosting of DAergic tone. Chronic pain and depression are often accompanied by hippocampal maladaptation characterized by decreased volume, reduced neurogenesis, and disrupted synaptic plasticity 13, 17, 52. For example, the presynaptic buttons and the frequency facilitation at the CA3-CA1 synapse were significantly impaired in spare nerve injury-induced neuropathic pain 53. Chronic stress impaired the AMPA receptor-dependent synaptic transmission in the hippocampus 54. We speculated that VTA^DA^ projection may modulate synaptic transmission and neuronal excitability in the vHPC by rescuing these deficits (Figure 10, right). In the susceptible mice, pyramidal neurons express more D1R than D2R, while D2R is the dominant type in GABAergic neurons. Phasic stimulation may cause direct excitation and inhibition of pyramidal and GABAergic neurons, respectively. The latter response can cause disinhibition of pyramidal neurons. These direct excitation and indirect disinhibition together may underlie the anti-depressant phenotype of phasic stimulation of the VTA^DA^-vHPC pathway. Administration of either D1R or D2R antagonists probably destroys the bipartite effects and results in depression.

Our results also indicated an equal involvement of D1R and D2R in maintaining the emotional balance in the resilient group. According to the Western blotting results, although the D1R level was indiscriminate among the three groups, the D2R level was also significantly upregulated in the resilient mice. Such upregulation may cause more receptor accumulation in GABAergic neurons due to its dominant expression propensity (Figure 10, middle). With a mechanism similar to that of boosting DAergic tone in the susceptible group, the direct D1R and indirect D2R effects in the vHPC may function together to cause resilience. Thus, the nuanced adaptation of D2R in the vHPC might contribute to the resilient phenotype after chronic pain. The significance of D2R has been proven in brain disorders such as schizophrenia, Parkinson's disease, and drug addiction, and it is the primary target for disease medications 44, 45, 55. The plastic changes and the role of hippocampal interneurons have also been highlighted in terms of resilience and vulnerability to different stressors 30. Our results suggested that a putative D2R-mediated plasticity of interneurons may contribute to depression resilience after neuropathic pain.

Besides the plastic changes and the delicate roles of D1R and D2R in the cuff model, the pharmacological results uncovered a leading role of D1R in maintaining normal emotion. This could be due to the relatively low level of D2R in the vHPC in naïve state, dampening the role of the indirect effect and leaving the direct one dominant (Figure 10, left). A previous study has demonstrated that phasic photostimulation of VTA^DA^ inputs increased the Schaffer collateral-evoked depolarization in CA1 pyramidal neurons by directly enhancing excitatory synaptic transmission through activation of D1-type receptors 56. Given the classical excitatory function of D1-type receptors, the dominant D1R over D2R expression in the pyramidal neurons of vCA1 and vCA3 may function similarly to maintain the synaptic transmission and mood balance. Caution should be exercised that this regulation might be independent of VTA^DA^ projection. As we observed the null behavioral influence following photostimulation. And another study reported a nonnegligible function of LC^DA^ projection that could corelease DA with norepinephrine in the hippocampus 57. Therefore, it is unambiguously clear that further research is needed to clarify the feature of synaptic transmission of the VTA^DA^-vHPC pathway and the role of D1R- or D2R-expressing vHPC neurons by discriminating differential chronic pain conditions.

## Conclusion

The VTA-HPC pathway has long been overlooked. Recent neural tracing and modulating strategies have delineated the anatomical feature and function of the VTA^DA^-dHPC pathway in learning and memory. We defined a medial dominant VTA^DA^ projection to vHPC, which was hypoactive under the comorbid depression of chronic pain whereas maintained normalcy in the resilient pain. Reinforcing the pathway efficiently reversed the depression without affecting pain in a parallel D1 and D2 receptor-dependent manner. Our results depict the anatomical properties and the role of the VTA-vHPC pathway in chronic pain and provide insights into a connectivity-based strategy in the treatment of comorbid depression.

## Methods

*Experimental animals.* Male wild-type (C57BL/6J) and transgenic mice (TH-Cre, GAD67-GFP, D1R-tdTomato) aged 8-10 weeks were obtained from the Laboratory Animal Center of the Fourth Military Medical University or the Jackson Laboratory. D2R-EGFP mice were a kind gift from the Institute of Health Sciences, Shanghai Institute of Life Science, Chinese Academy of Science. Mice were housed on a 12 h light/dark cycle (light on at 08:00) with water and food *ad libitum.* The room temperature was kept at 22-25 °C with 50 ± 10% humidity. All experimental procedures complied with the Guide from the Animal Use and Care Committee for Research and Education of the Fourth Military Medical University (Xi'an, China).

*Nerve cuffing model*. The cuff model was established to investigate the pain- and depression-like mechanisms as described previously 33, 58. All mice were anesthetized using 2% isoflurane (R510-22-10, RWD, Shenzhen, China) and placed on a 37 °C heating pad during the surgery. The surgical area was prepared from the hip to the knee on the left, and the incision was made parallel to the sciatic nerve. The main trunk of the sciatic nerve was exposed and separated from the surrounding connective tissue. A 2 mm long PE-20 polyethylene tube (62325; RWD) was placed around the nerve. For the sham mice, the sciatic nerve was exposed similarly without any nerve damage. The incision was sutured and disinfected with iodophor.

*Stereotaxic injection.* All stereotaxic surgeries were performed under general anesthesia with 2% isoflurane. The anesthetized mice were positioned in a stereotaxic instrument (Narishige Scientific Instrument Lab, Tokyo, Japan) and were injected with tracers or viruses through a glass micropipette attached to a 1 μL microsyringe (Hamilton, Reno, NV, USA). All tracers and viruses (see Table S2) were injected into the VTA (anterior-posterior (AP): -3.35 mm, medial-lateral (ML): ±0.45 mm, dorsal-ventral (DV): -4.45 mm), vHPC (AP: -3.10 mm, ML: ±3.15 mm, DV: -4.05 mm), mPFC (AP: 1.93 mm, ML: ±0.30 mm, DV: -2.35 mm), NAc (AP: 1.40 mm, ML: ±1.00 mm, DV: -4.45 mm) at a rate of 15 nL min^-1^. The micropipette was maintained in the injection site for 10 min at the end of infusion to avoid overflow.

*Optic fiber or guide cannula implantation.* For optogenetic or pharmacological manipulation, the optic fiber cannula (NA 0.37, 200 μm; Newdoon, Hangzhou, China) or guide cannula (RWD) was implanted over 200 μm in the bilateral vHPC 3 weeks after virus injection and was fixed on the skull with bone screws, super glue, and dental cement.

*Optogenetics.* Optic fibers were connected to a 473 nm blue laser generator. For all *in vivo* behavioral experiments, mice were given either low-frequency tonic (10 ms, 1 Hz, continuously) or high-frequency phasic (10 ms, 20 Hz, 20 flashes, every 2 s) light stimulations 20, 42, 59. The power of the blue laser was calibrated to 6 mW.

*Drugs and pharmacology.* In chemogenetic modulation, four weeks after viral injection, an intraperitoneal injection of CNO (2 mg/kg, diluted with 0.9% saline; C8032, Sigma-Aldrich, St. Louis, MO, USA) was given 40 min ahead of the behavior tests. In pharmacological manipulation, the guide cannula was implanted into the bilateral vHPC to allow intracranial administration of drugs and delivery of a 473 nm laser. The D1R antagonist SCH23390 hydrochloride (0.1 μg, 0.05 μg/side; D054, Sigma-Aldrich) or D2R antagonist sulpiride (0.2 μg, 0.1 μg/side; S8010, Sigma-Aldrich) was delivered at the rate of 0.1 μL/min (total volume of 0.3 μL per side) 42. The vehicle (same amount of 0.9% saline) was used as a control. Laser stimulation and behavioral tests were performed 10 min after intracranial injection.

*Behavioral assays.* Behavioral tests were performed as previously described with minor modifications 33. The mice were habituated by handling for three days before behavioral assays. Experimenters were blinded to animal grouping and outcome assessment. Behavioral tests were conducted between 9 a.m. and 6 p.m., and the equipment was cleaned with 70% ethanol between every two animals.

Mechanical allodynia. Mice were habituated in the transparent plastic boxes (7 × 7 × 10 cm) with a metal grid (1 × 1 cm) floor for 40 min before testing. A series of nine von Frey filaments (vF, Stoelting, Kiel, WI, USA) (0.008, 0.02, 0.04, 0.16, 0.4, 0.6, 1, 1.4, and 2 g) with different bending forces (0.078, 0.196, 0.392, 1.568, 3.92, 5.88, 9.8, 13.72, and 19.6 mN) were gently and perpendicularly applied to the plantar surface of bilateral hind paws until the filament bent (for ≤ 3 s). Responses such as licking, flinching, shaking, and paw withdrawal were regarded as positive. The minimum bending force evoking 3 positive withdrawals in 5 trials was defined as the paw withdrawal threshold.

Open field test. The OFT was conducted to evaluate the general locomotor, exploration, and anxiety in animals. The mice were placed in the center of an open field arena (50 cm × 50 cm × 40 cm), and their movements were recorded for 15 min using a video camera. The total travel distance, central time, and central distance percentage were analyzed by the Anymaze video-tracking software (Stoelting, Wood Dale, IL, USA).

Elevated plus maze. The EPM test was used to evaluate the anxiety-related behaviors in mice. The apparatus comprises two opposing closed arms (60 cm × 5 cm × 15 cm) and two open arms (60 cm × 5 cm). Each mouse was placed in the central platform with its head directed toward the same open arm and allowed to explore the maze for 5 min. The time spent in and entry percentage into open arms were calculated.

Tail suspension test. Mice were suspended by the tail using tape for 6 min. The approximate distance between the mouse's nose and the apparatus floor is 20-25 cm. The mice could not see other objects or touch the apparatus during the assay. The activity was monitored by a video, and immobility was defined as when the mouse did not want to put any effort into trying to escape. The immobile time of mice during the last 5 min was analyzed.

Novelty suppressed feeding. The NSF was used as a measure of depression-like status. Following 30 h of food deprivation, the mice were placed in the corner of a white square arena (50 cm × 50 cm × 40 cm) containing a food pellet in the center. The latency to the first feeding, defined as biting the pellet with forepaws, was recorded.

Forced swim test. Similar to the TST, the FST is usually used to assess depression-like behaviors. Mice were placed in a plastic cylinder (height, 17.5 cm; diameter, 12.5 cm) filled with 11.5 cm of water (23-25 °C) for 6 min. The water depth was set to guarantee that the mouse's tail could not touch the bottom of the apparatus. The movement was recorded, and the immobile time during the last 5 min was calculated to evaluate the stress-coping ability.

*Immunofluorescent histochemical staining.* The procedures were performed as previously described 60. In brief, mice were deeply anesthetized with overdosed isoflurane and then transcardially perfused with 0.01 M phosphate-buffered saline (PBS, pH 7.4) followed by 0.1 M phosphate buffer (PB, pH 7.4) containing 4% paraformaldehyde. The whole brains were removed and cryoprotected in 30% (w/v) sucrose in PB for 48 h. Brain blocks containing VTA, vHPC, mPFC, or NAc were cut into 25 μm-thick sections on a cryostat (CM1800, Leica, Heidelberg, Germany) and were collected into 6 series in dishes. After being blocked in 10% normal donkey serum for half an hour at room temperature, the sections were incubated with varied antibodies in 0.01 M PBS containing 0.3% Triton X-100, 0.1% λ-carrageenan, and 1% donkey serum, 0.02% NaN_3_ (pH 7.4) for immunostaining. Detailed antibody information is listed in Table S3. The staining was observed under a confocal laser scanning microscope (FV-1000, Olympus, Tokyo, Japan). For FOS detection in the chemogenetic experiment, mice were intraperitoneally injected with CNO (2 mg/kg) 2 h before perfusion. To check the position of the optic and guide cannula, DAPI (1:1000; sc-3598, Santa Cruz, Dallas, TX, USA) was used to counterstain the section for 10 min to show the vHPC profile. Cell counting for FOS-immunoreactive (ir), TH-ir, and FOS/TH double-labeled neurons in the VTA (containing mVTA and lVTA) was conducted in the sham, resilient, and susceptible mice. Five sections containing VTA in one series for each mouse were included in counting, and every group contained three mice for analysis.

*Fluorescence in situ hybridization.* FISH was performed as previously described 60. The D1R-tdTomato, D2R-EGFP, GAD67-GFP, and C57BL/6J mice were perfused, and the brains were dissected and serially cut into 30 μm thick sections. The sections containing NAc or vHPC were used for FISH and immunofluorescent staining. In brief, the sections were treated with 2% H_2_O_2_ in 0.1 M of PB for 10 min. After rinsing, the sections were incubated in 0.3% Triton-X100 for 20 min. The sections were then incubated for 10 min in an acetylation solution containing 0.25% (v/v) acetic anhydride in 0.1 M triethanolamine. After rinsing, the sections were prehybridized for 1 h at 60 °C. D1R or D2R riboprobes were subsequently added to the hybridization system at a final concentration of 1 μg/mL and incubated at 60 °C for 20 h. After washing, the hybridized sections were incubated with 20 μg/mL ribonuclease for 30 min at 37 °C. Then, sections were incubated with various antibodies for visualization. In addition, to amplify the D1R mRNA or D2R mRNA hybridization signals we also performed the biotinylated tyramine (BT)-glucose oxidase (GO) amplification method for 30 min. Detailed information on antibodies is listed in Table S3.

*Western blotting.* After anesthesia, mice were sacrificed by decapitation. The brain was quickly removed, and the VTA and vHPC areas were collected. The tissue was homogenized with an ultrasound homogenizer in a precooled RIPA buffer. The homogenates were centrifuged at 12,000×g for 15 min at 4 °C. The protein content was detected using the bicinchoninic acid (BCA) kit (C05-02001, Bioss, Beijing, China). The protein samples were heated at 99 °C for 5 min and loaded on a 10% SDS/polyacrylamide gel and transferred to polyvinylidene fluoride membranes (Immobilon-P, Millipore, Hayward, CA, USA). The membranes were blocked with 5% (w/w) non-fat dried milk and incubated with primary and secondary antibodies (information see Table S3**)**. An enhanced chemiluminescence kit (WBKLS0050, Millipore) was used to detect immunoreactive protein bands. The intensity of the band was analyzed by the Image Lab software (BioRad, Hercules, CA, USA) to calculate the ratio of target protein to internal control (β-actin).

*Ex vivo electrophysiology.* The procedures were conducted as in previous publications 33, 61. After being anesthetized, mice subjected to TMR injection in the bilateral vHPC in C57BL/6J mice or AAV2/R-EF1α-DIO-mCherry injection in TH-Cre mice were quickly decapitated. The brain was removed to make coronal VTA slices (300 μm) in a vibrating microtome (VT 1200s, Leica) at 0-4 °C. The slices were transferred into oxygenated (95% O_2_ plus 5% CO_2_) artificial cerebrospinal fluid (ACSF) containing 124 mM NaCl, 2.5 mM KCl, 1 mM NaH_2_PO_4_, 25 mM NaHCO_3_, 1 mM MgSO_4_, 2 mM CaCl_2_, 10 mM glucose. After 90 min of recovery, slices were transferred to a recording chamber. The TMR- or mCherry-labeled neurons were visualized under a microscope with infrared-differential interference contrast or fluorescent optics video microscopy (BX51WI, Olympus). In most cases, patch pipettes (3-5 MΩ) were filled with a solution containing 124 mM K-gluconate, 10 mM HEPES, 10 mM phosphocreatine disodium, 1 mM MgCl_2_, 5 mM NaCl, 2 mM MgATP, 0.2 mM EGTA, and 0.1 mM Na_3_GTP (adjusted to pH 7.2 with KOH, 290-300 mOsmol). For sIPSC recording (Figure 4K-M), a cesium-based pipette solution was used: 112 mM Cs-gluconate, 10 mM HEPES, 5 mM TEA-Cl, 5 mM QX-314, 3.7 mM NaCl, 2 mM MgATP, 0.2 mM EGTA, and 0.3 mM Na_3_GTP. All recordings were performed with an Axon 700B amplifier (Axon Instruments, Foster City, CA, USA). The Clampex (v.10.6, Axon Instruments) and Clampfit software (v.10.6, Axon Instruments) were utilized for data acquisition and analysis. In voltage clamp mode, sEPSC was recorded for 3 min at a holding potential of -60 mV and sIPSC at 0 mV. In current clamp mode, the rheobase of TMR-labeled neurons was detected with the supra-threshold depolarized currents of 0-200 pA (30 ms duration) delivered in increments of 5 pA. The firing of TMR-labeled neurons was also detected by recording action potentials in response to depolarized currents of 0-100 pA (400 ms duration) in increments of 25 pA. Biocytin (0.2%; Sigma-Aldrich) was included in the pipette solution to identify the recorded neurons. To detect the efficacy of the chemogenetic virus, the effect of bath application of CNO (10 μM) on the discharge of hM3Dq-expressing neurons was conducted with membrane potential clamped around -70 mV (Figure 5E). To detect the effectiveness of the optogenetic virus, the 15 pA current was injected into the ChR2-expressing neurons with or without blue laser stimulation (473 nm, 2 s, 1 Hz, or 20 Hz) through a ×40 objective lens (Figure 7E). Data were discarded if the access resistance changed > 15% during the experiment. Data were filtered at 1 kHz and digitized at 10 kHz. All data were analyzed using Mini Analysis (Synaptosoft, Fort Lee, NJ, USA) and Clampfit software.

*Statistics.* GraphPad Prism 8 was used for the statistical analysis. Statistical significance was assessed by unpaired Student's *t*-test, 1-way and 2-way ANOVA, and 2-way repeated-measures (RM) ANOVA or their corresponding nonparametric tests. Tukey's post hoc test, Šidák's post hoc, and other post hoc corresponding to nonparametric tests were applied when ANOVA showed a significant main effect. All data are presented as the mean ± standard error of the mean (SEM). Statistical significance was indicated as *P < 0.05, **P < 0.01, ***P < 0.001, and ****P < 0.0001.

## Supplementary Material

Supplementary figures and tables.

Supplementary statistics.

## Abbreviations

ACC: anterior cingulate cortex
BNST: bed nucleus of the stria terminalis
CNO: clozapine N-oxide
D1R: dopamine 1 receptor
D2R: dopamine 2 receptor
DAergic: dopaminergic
dHPC: dorsal hippocampus
DREADDs: designer receptors exclusively activated by designer drugs
EPM: elevated plus maze
FG: Fluoro-Gold
FISH: fluorescence *in situ* hybridization
FST: forced swim test
GAD: glutamate decarboxylase
HPC: hippocampus
LC: locus coeruleus
LTP: long-term potentiation
mPFC: medial prefrontal cortex
NAc: nucleus accumbens
NAcLat: lateral shell of NAc
NAcMed: medial shell of NAc
NSF: novelty suppressed feeding
OFT: open field test
PFC: prefrontal cortex
sEPSC: spontaneous excitatory postsynaptic current
sIPSC: spontaneous inhibitory postsynaptic current
SN: substantia nigra
TH: tyrosine hydroxylase
TMR: tetramethyl rhodamine
TST: tail suspension test
vHPC: ventral hippocampus
VTA: ventral tegmental area
