# Supplementary material for: Modulation of comorbid depression of neuropathic pain by dopamine input from VTA to the ventral hippocampus
Source: Theranostics. 2025 Mar 10;15(9):4101–23. doi: 10.7150/thno.104394 (PMC11980670; doi:10.7150/thno.104394)
Supplement: Supplementary file 1 — Supplementary figures and tables. [file thnov15p4101s1.pdf]

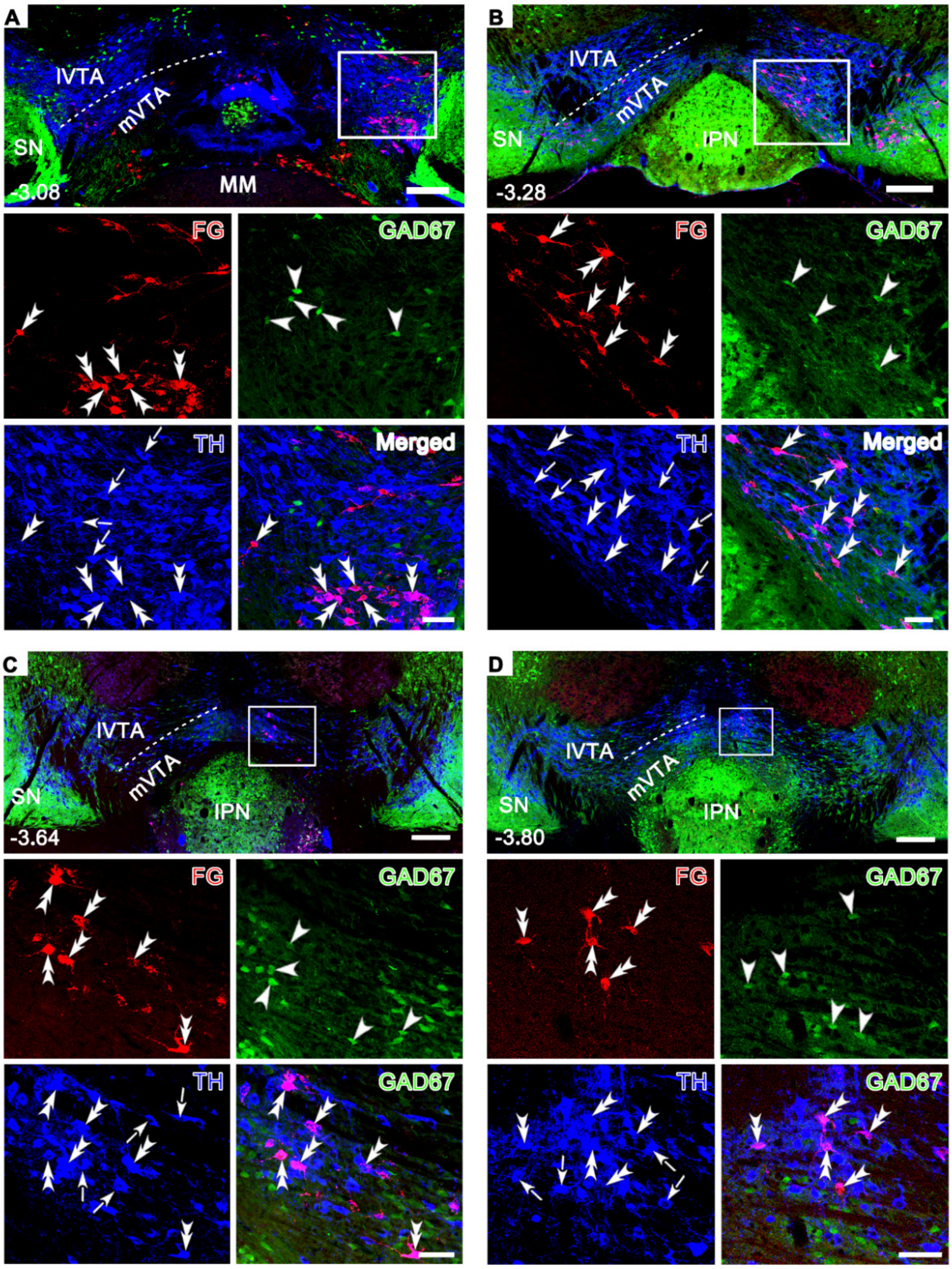

Figure S1. Distribution of FG-labeled neurons along the rostrocaudal sections of VTA. (A-D) Triple immunofluorescent images showing FG-labeled projection neurons (red), GFP-labeled GABAergic neurons (green), and TH-labeled DAergic neurons (blue) in the rostrocaudal VTA. Rostral level: Bregma -3.08 mm **(A)**. Rostral-middle level: Bregma -3.28 mm **(B)**. Middle-caudal level: Bregma -3.64, -3.80 mm **(C** and **D)**. The white rectangular areas are amplified below, with double arrowheads indicating FG/TH double-labeled neurons, arrows indicating TH single-labeled neurons, and arrowheads indicating GFP single-labeled neurons. Scale bar: 200 μm (upper panels in A-D), 50 μm (lower panels in A-D). IPN, interpeduncular nucleus; MM, medial mamillary nucleus, medial part; SN, substantia nigra.

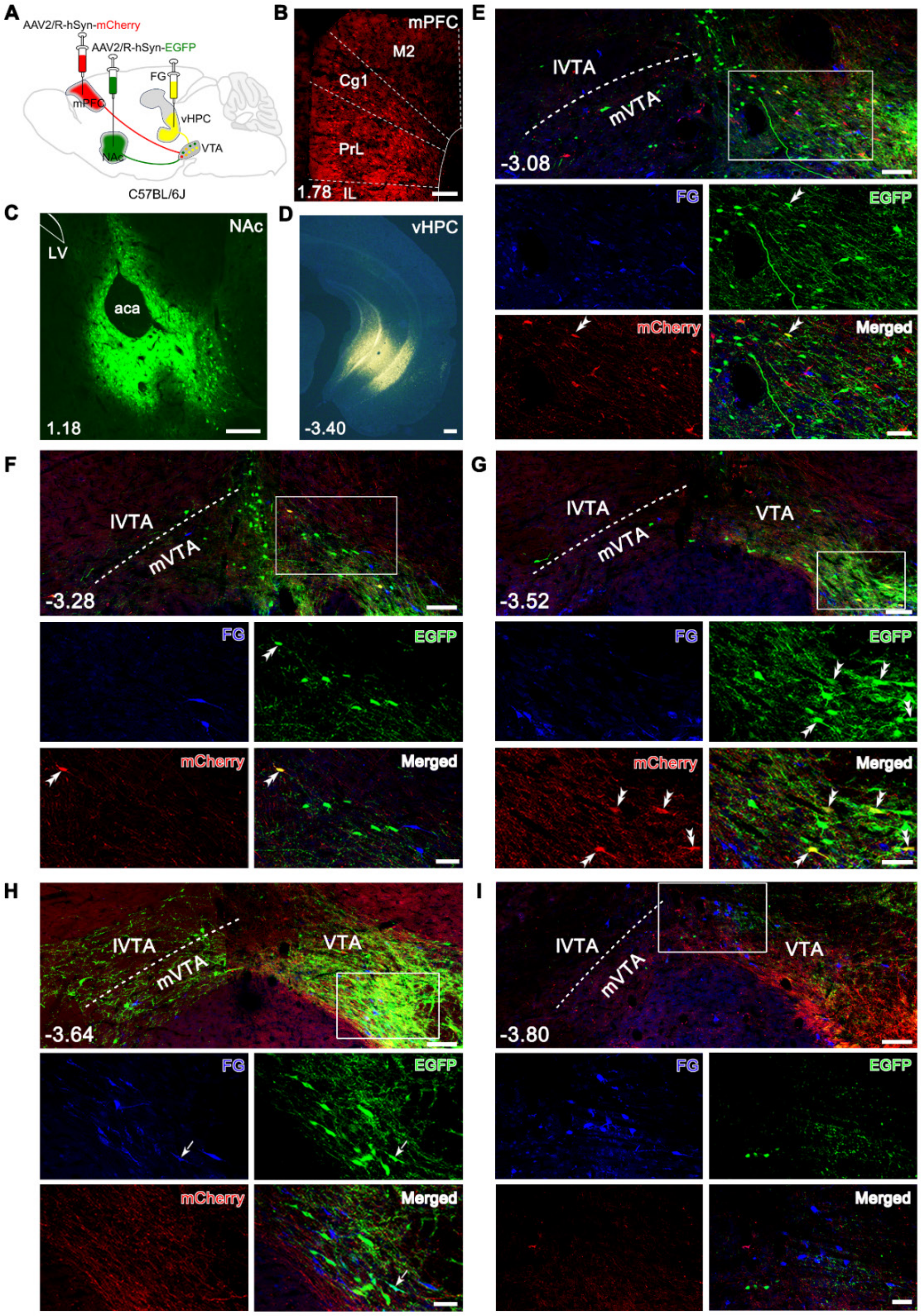

Figure S2. Collateral projecting features of the VTA-vHPC pathway compared with VTA-mPFC and VTA-NAc projections. (A) Schematic diagram for injection of varied retrograde tracers. AAV2/R-hSyn-mCherry, AAV2/R-hSyn-EGFP, and FG were respectively injected into the right side of the mPFC, NAc, and vHPC in C57BL/6J mice. **(B-D)** Representative coronal sections showing the injection sites. Scale bar: 200 μm. **(E-I)** Images showing the distribution of EGFP, mCherry, and FG-labeled neurons in the VTA at different segments (Bregma: -3.08, -3.28, -3.52, -3.64, and -3.80 mm). White rectangular areas are amplified below with double arrowheads indicating EGFP/mCherry double-labeled neurons, and arrows EGFP/FG double-labeled neurons. Scale bar: 100 μm (upper panels in E-I), 50 μm (lower panels in E-I). Cg1, cingulate cortex, area 1; IL, infralimbic cortex; M2, secondary motor cortex; PrL, prelimbic cortex.

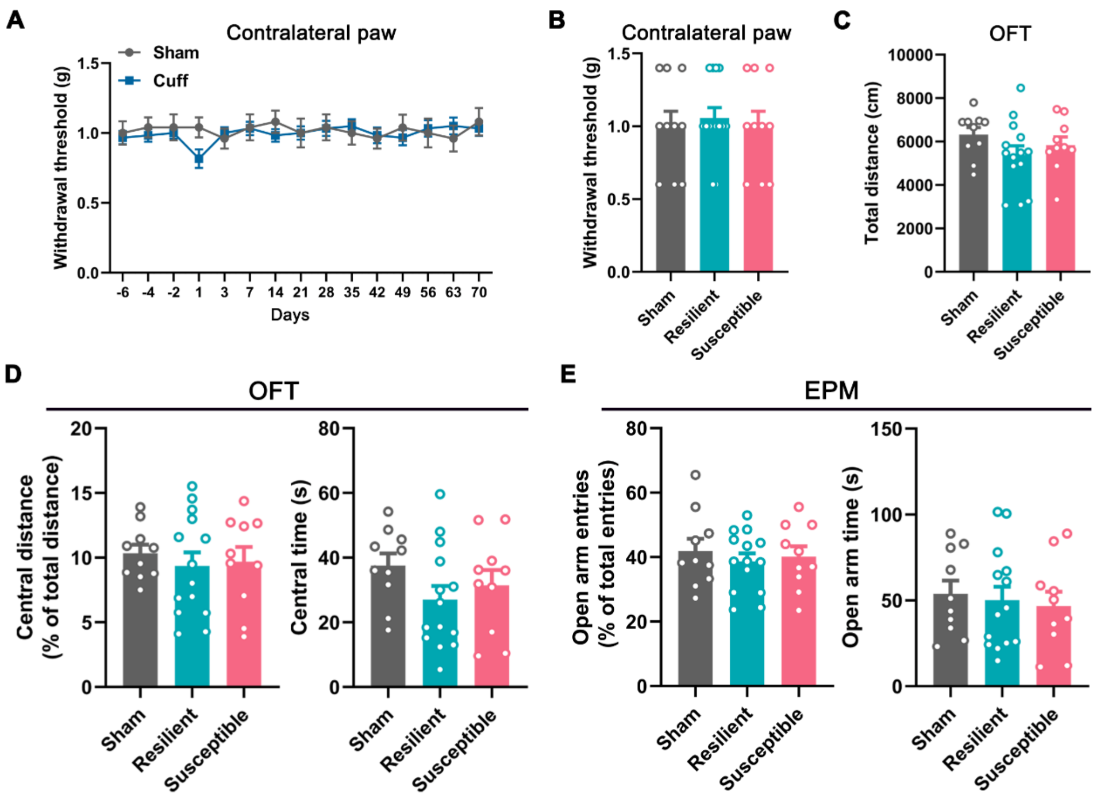

Figure S3. Contralateral mechanical sensitivity and animal performance in the OFT and EPM in the cuff model. (A) Long-term observation of the mechanical threshold in the contralateral hind paw. The cuff operation did not alter the mechanical pain threshold in the contralateral hind paw. n = 10 in the sham group, n = 24 in the cuff group. 2-way RM ANOVA with Šidák's post hoc test. **(B)** Resilient and susceptible mice had no mechanical pain in the contralateral hind paw at POW 8. n = 10-14. Kruskal-Wallis test with Dunn's post hoc test. **(C)** The total distance traveled in the OFT was similar in the sham, resilient and susceptible groups at POW 8. n = 10-14. 1-way ANOVA with Tukey's post hoc test. **(D)** Percentage of central distance traveled and central time spent in the OFT did not alter among different groups. n = 10-14. 1-way ANOVA with Tukey's post hoc test. **(E)** Percentage of open arm entries and open arm time in the EPM were indiscriminate in three groups. n = 10-14. 1-way ANOVA with Tukey's post hoc test.

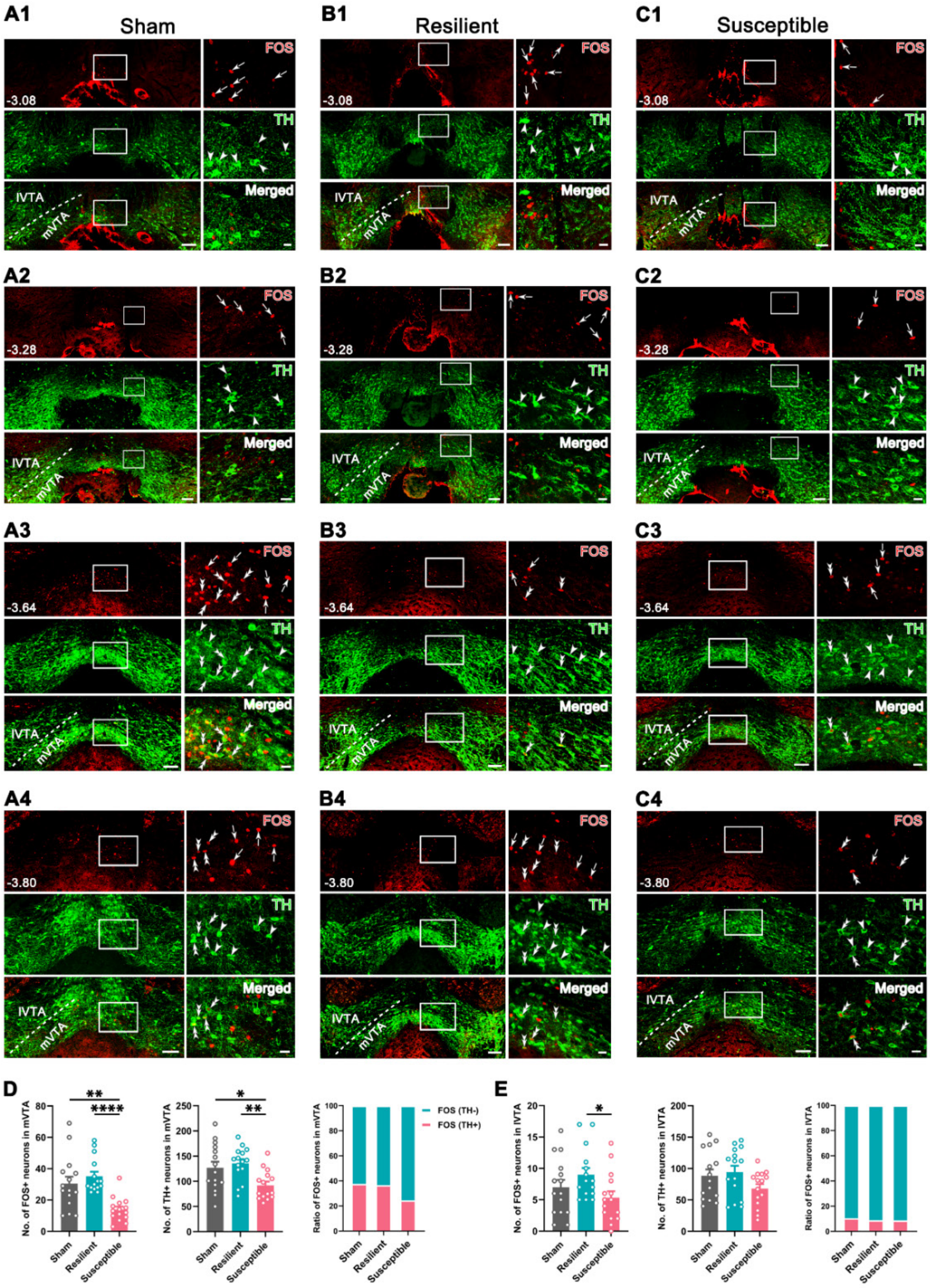

Figure S4. Distribution and number of FOS and TH positive neurons in the VTA in three groups. (A1-C4) Typical pictures showing double immunofluorescent staining of FOS (red) and TH (green) in the rostral to caudal VTA (Bregma -3.08, -3.28, -3.64, and -3.80 mm). The white rectangle is enlarged on the right, with double arrowheads indicating FOS/TH double-labeled neurons, arrows indicating FOS single-labeled neurons, and arrowheads indicating TH single-labeled neurons. The dashed line indicates the location of the mVTA and lVTA. Scale bar: 100 μm (left panels in A1-C4), 20 μm (right panels in A1-C4). **(D** and **E)** Comparison of the number of FOS positive neurons, TH positive neurons, and the ration of FOS expression in the mVTA and lVTA. Susceptible mice showed a more prominently decreased number of FOS and TH positive neurons in the mVTA than in the lVTA. The ratio of FOS/TH double-labeled neurons to FOS-labeled neurons

in the mVTA was also lowered in the susceptible mice. n = 3 mice (15 sections) per group. *P < 0.05, **P < 0.01, ****P < 0.0001, FOS in the mVTA and lVTA: Kruskal-Wallis test with Dunn's post hoc test; TH in the mVTA and lVTA: 1-way ANOVA with Tukey's post hoc test.

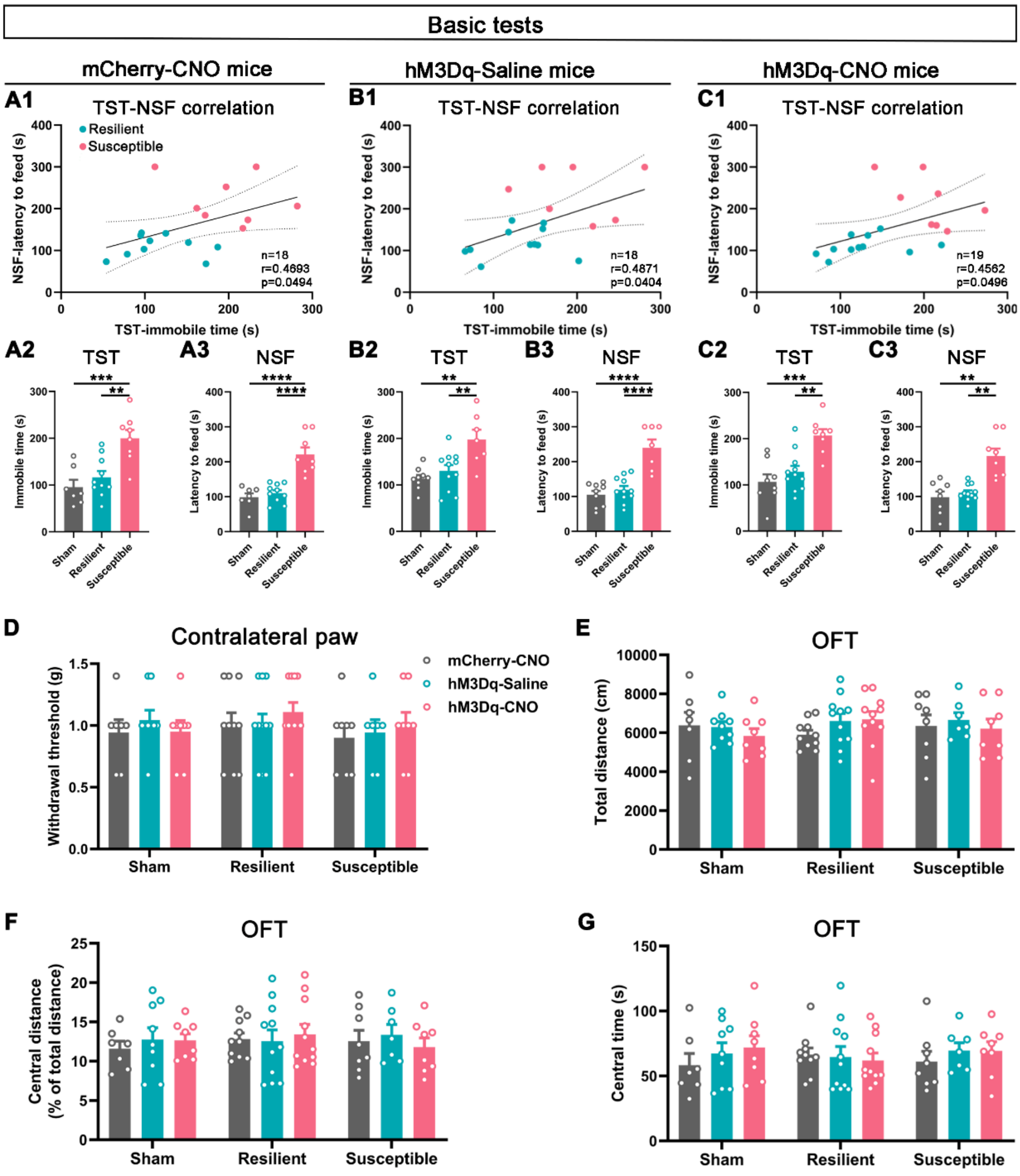

Figure S5. Animal classification in chemogenetic experiments and the activating effect on contralateral pain, locomotor activities, and anxious status of the three groups. (A-C) Basic tests by the TST and NSF to classify the cuff mice at POW 8 into the resilient and susceptible groups for subsequent mCherry-CNO, hM3Dq-Saline, and hM3Dq-CNO manipulations. **(A1-C1)** Correlation analysis of cuff mice performance in the TST and NSF in each group. n = 18-19. Pearson's correlation test. **(A2-C2)** Comparison of the TST immobile time in three groups, showing the increased value in the susceptible group. n = 7-11. **P < 0.01, ***P < 0.001, by 1-way ANOVA with Tukey's post hoc test. **(A3-C3)** Increased latency to feed of susceptible mice in the NSF. n = 7-11. **P < 0.01, ****P < 0.0001, by 1-way ANOVA with Tukey's post hoc test (A3 and B3) and

Welch's ANOVA test with Tamhane's T2 post hoc test (C3). **(D)** Chemogenetic activation of VTA-vHPC projection neurons did not affect the mechanical sensitivity in the contralateral hind paw in the sham, resilient, and susceptible groups. *n* = 7-11. 2-way ANOVA with Šidák's post hoc test. **(E-G)** Chemogenetic activation of vHPC-projecting VTA neurons did not affect the total distance, central distance percentage, and central time in the OFT in the three groups. *n* = 7-11. 2-way ANOVA with Šidák's post hoc test.

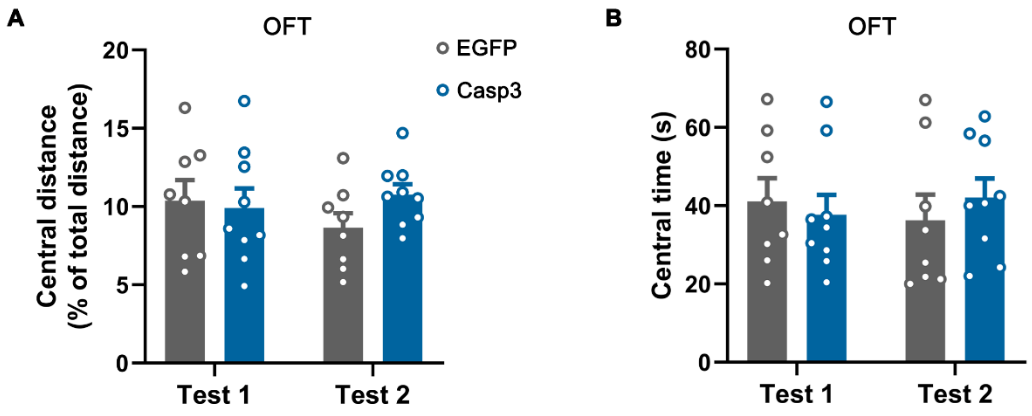

Figure S6. No effect of ablation of VTA-vHPC projection neurons on animal performance in the OFT. (A and **B)** Percentage of central distance traveled and central time spent in the OFT were similar between the EGFP group and Casp3 group in Test 1 and Test 2. *n* = 8-9. 2-way RM ANOVA with Šidák's post hoc test.

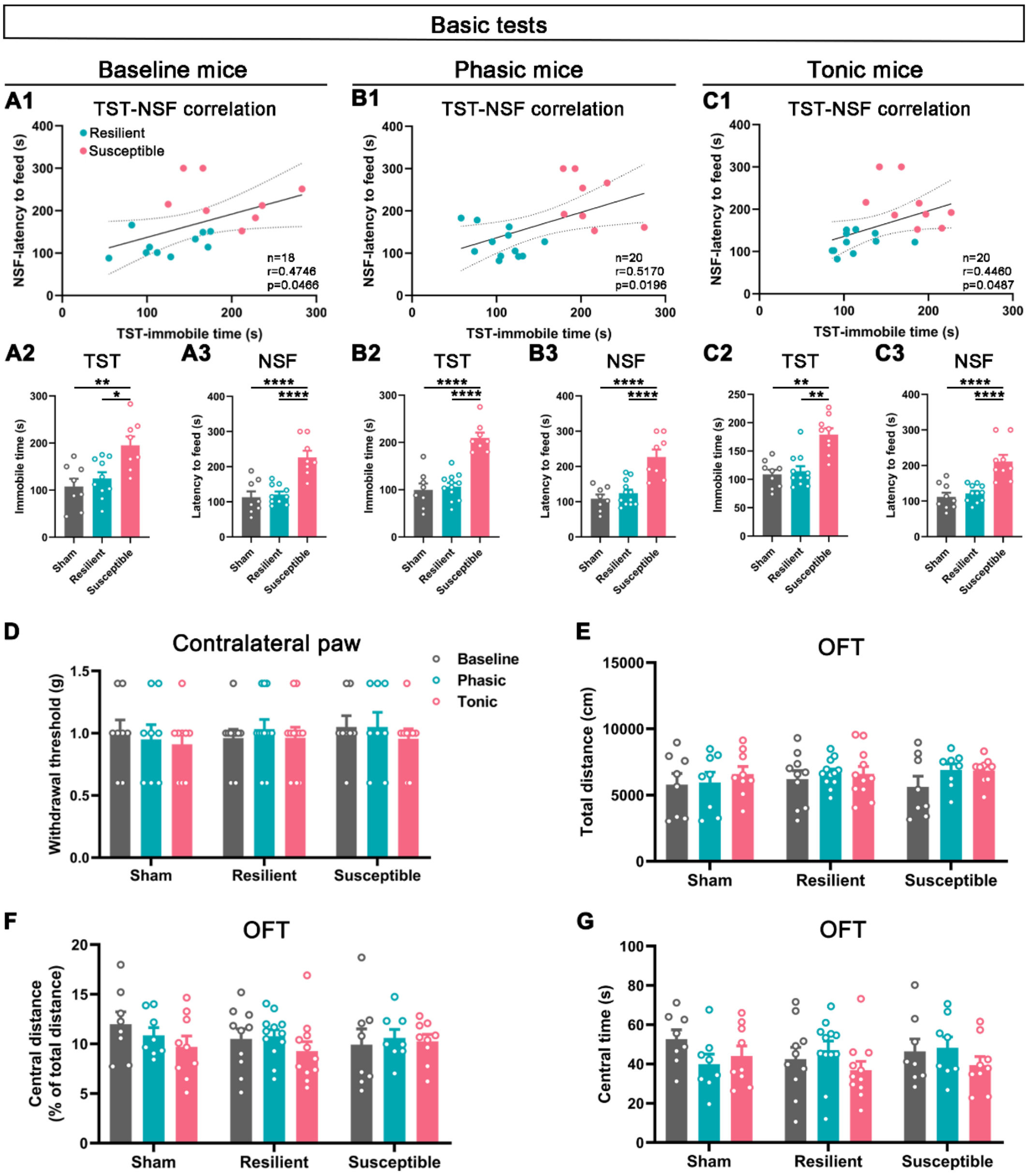

Figure S7. Animal classification in optogenetic experiments and the activating effect on contralateral pain, locomotor activities, and anxious status in mice. (A-C) Cuff mice were classified into the resilient and susceptible groups according to animal performance in the basic tests of TST and NSF. **(A1-C1)** Correlation analysis of animal performance in the TST and NSF in cuff mice. n = 18-20. Pearson's correlation test. **(A2-C2)** Increased immobile time in the TST in the susceptible mice. n = 8-12. *$P < 0.05$, **$P < 0.01$, ****$P < 0.0001$, by 1-way ANOVA with Tukey's post hoc test (A2 and B2) and Kruskal-Wallis test with Dunn's post hoc test (C2). **(A3-C3)** Increased latency to feed in the NSF in the susceptible mice. n = 8-12. ****$P < 0.0001$, by 1-way ANOVA with Tukey's post hoc test. **(D)** Phasic or tonic photostimulation of VTA^{DA}-vHPC projection did not affect contralateral mechanical sensitivity. n = 8-12. 2-way ANOVA with Šidák's post hoc test. **(E-G)** Total distance, central distance percentage, and central time in the OFT were indiscriminate among the sham, resilient and susceptible groups with or without phasic and tonic stimulation, respectively. n = 8-12. 2-way ANOVA with Šidák's post hoc test.

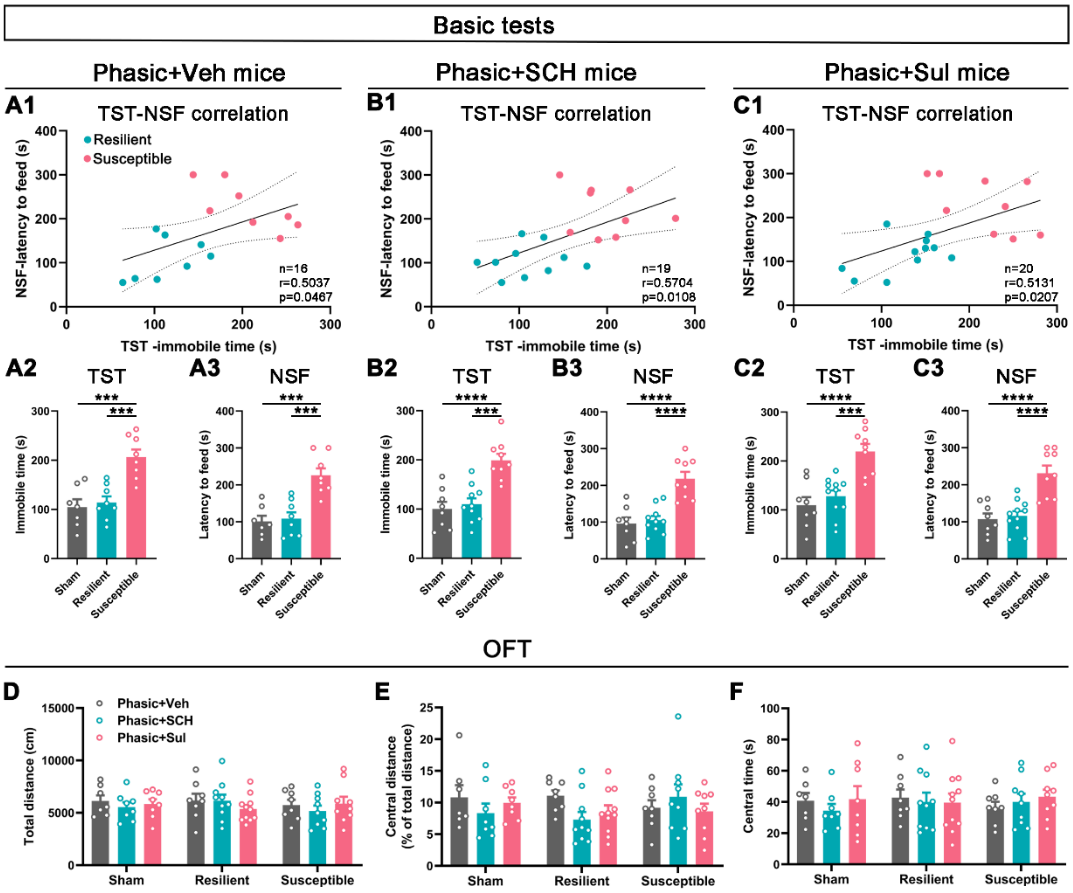

Figure S8. Animal classification in pharmacological experiments and the effect of drug delivery on locomotor activities and anxious status in mice. (A-C) Cuff mice were classified into the resilient and susceptible groups according to animal manifestations in the basic tests of TST and NSF. **(A1-C1)** Correlation analysis of animal performance in the TST and NSF in cuff mice. *n* = 16-20. Pearson's correlation test. **(A2-C2)** Increased immobile time in the TST in the susceptible mice. *n* = 7-11. ****P* < 0.001, *****P* < 0.0001, by 1-way ANOVA with Tukey's post hoc test. **(A3-C3)** Increased latency to feed in the NSF in the susceptible mice. *n* = 7-11. ****P* < 0.001, *****P* < 0.0001, by 1-way ANOVA with Tukey's post hoc test. **(D-F)** The effect of phasic stimulation and pharmacological intervention on animal performance in the OFT. SCH23390 (SCH, D1R antagonist) or sulpiride (Sul, D2R antagonist) was stereotactically applied to the vHPC 10 minutes before of photostimulation. Total distance, central distance percentage, and central time in the OFT were indiscriminate in the sham, resilient and susceptible groups after the treatments. *n* = 7-11. 2-way ANOVA with Šidák's post hoc test.

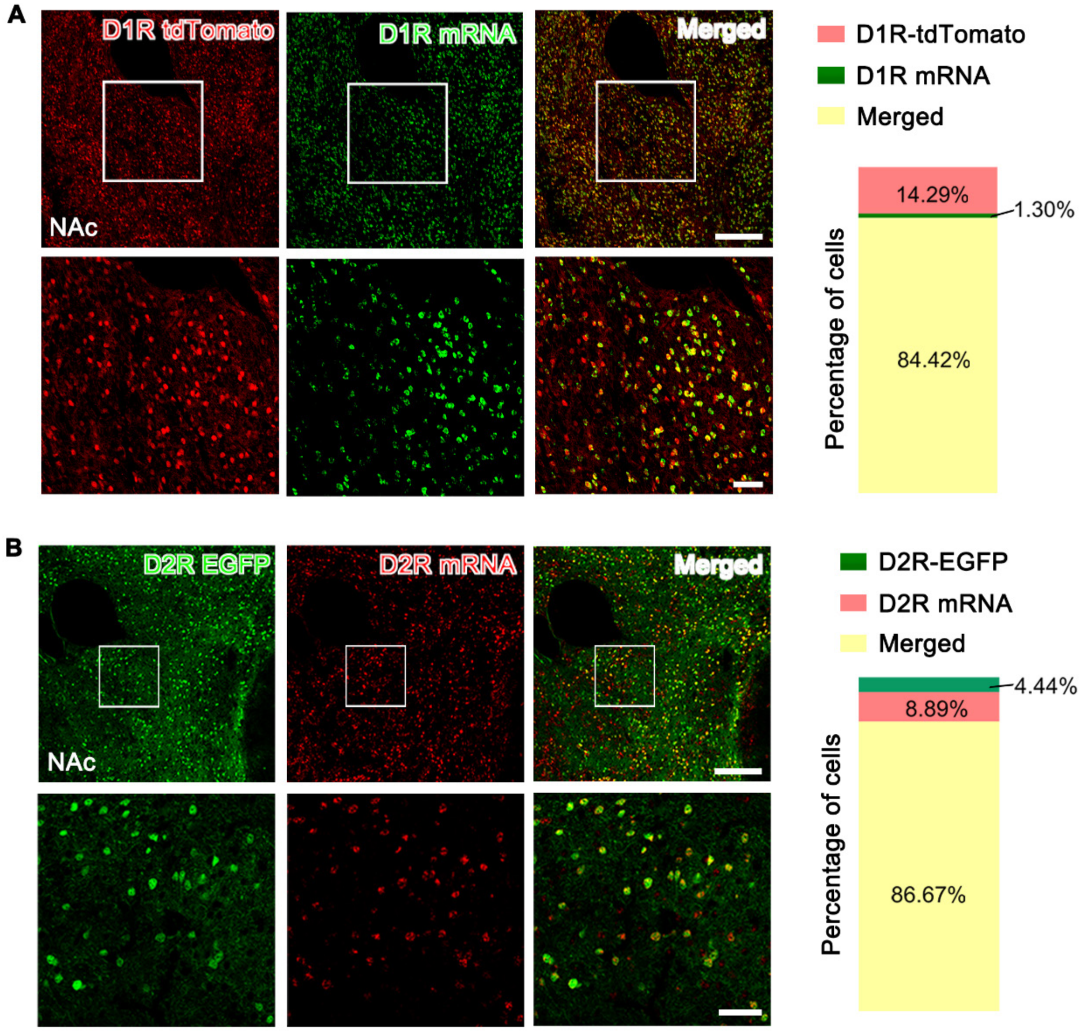

Figure S9. FISH with D1R and D2R probes in D1R-tdTomato and D2R-EGFP mice to verify the probe specificity. Left, typical images showing D1R mRNA/tdTomato or D2R mRNA/EGFP double-labeled neurons in the NAc in D1R-tdTomato or D2R-EGFP mice. The white rectangular area is enlarged at the bottom. Scale bar: 200 μm (upper panels in A and B), 50 μm (lower panels in A and B). Right, illustration of the percentage of double-labeled neurons and single-labeled neurons.

Table S1. Number of FG-labeled and FG/TH double-labeled neurons in the VTA.

Mouse	Bregma (mm)	FG					FG/TH		
		Ipsi	Con	mVTA	lVTA	Total	mVTA	lVTA	Total
M1	-3.08	40	3	31	12	43	28	11	39
	-3.28	33	13	35	11	46	33	11	44
	-3.52	20	7	23	4	27	21	4	25
	-3.64	11	0	11	0	11	9	0	9
	-3.80	8	3	8	3	11	6	2	8
	Total	112	26	108	30	138	97	28	125
M2	-3.08	39	5	32	12	44	30	12	42
	-3.28	33	10	34	9	43	33	7	40
	-3.52	20	7	24	3	27	23	3	26
	-3.64	9	0	8	1	9	8	1	9
	-3.80	6	2	7	1	8	6	1	7
	Total	107	24	105	26	131	100	24	124
M3	-3.08	39	7	33	13	46	30	12	42
	-3.28	38	10	39	9	48	37	9	46
	-3.52	26	13	32	7	39	29	6	35
	-3.64	11	3	12	2	14	9	2	11
	-3.80	9	2	8	3	11	5	3	8
	Total	123	35	124	34	158	110	32	142

Ipsi: ipsilateral, Con: contralateral.

Table S2. Tracer and virus injections

Figure	Tracers and viruses	Target nuclei	Volume	Serial Number
Fig. 1A	FG (4%)	Right vHPC	80 nL	
Fig. 1E	AAV2/9-hSyn-DIO-synaptophysin-mCherry (2.21×10^{12} vg/mL)	Bilateral VTA	200 nL/side	PT-2755 (BrainVTA, Wuhan, China)
Fig. 4A	TMR (10%)	Bilateral vHPC	300 nL/side	
	AAV2/R-EF1α-DIO-mCherry (5.00×10^{12} vg/mL)	Bilateral vHPC	300 nL/side	PT-0013 (BrainVTA)
Fig. 5B	AAV2/9-Ef1α-DIO-hM3Dq-mCherry (2.21×10^{12} vg/mL)	Bilateral VTA	200 nL/side	PT-0042 (BrainVTA)
	AAV2/9-EF1α-DIO-mCherry (2.12×10^{12} vg/mL)	Bilateral VTA	200 nL/side	PT-0013 (BrainVTA)
	AAV2/R-hSyn-CRE (2.93×10^{12} vg/mL)	Bilateral vHPC	300 nL/side	PT-0136 (BrainVTA)
Fig. 6B	AAV2/9-EF1α-DIO-taCasp3-TEVp-P2A-EGFP (2.91×10^{12} vg/mL)	Bilateral VTA	200 nL/side	PT-1230 (BrainVTA)
	AAV2/9-EF1α-DIO-EGFP (2.36×10^{12} vg/mL)			PT-0842 (BrainVTA)
	AAV2/R-hSyn-CRE	Bilateral vHPC	300 nL/side	

	(2.93×10^{12} vg/mL)			
Fig. 7B and 8B	AAV2/9-Ef1α-DIO-hChR2(H134R)-mCherry (5.27×10^{12} vg/mL)	Bilateral VTA	200 nL/side	PT-0002 (BrainVTA)
Fig. S2	AAV2/R-hSyn-mCherry (5.01×10^{12} vg/mL)	Right mPFC	200 nL	BC-0023 (Brain Case, Shenzhen, China)
	AAV2/R-hSyn-EGFP (5.01×10^{12} vg/mL)	Right NAc	200 nL	BC-0020 (Brain Case)
	FG (4%)	Right vHPC	80 nL	

Table S3. Antibodies

Figure	Antigen	Primary antibodies	Secondary antibodies
Fig. 1D and S1	FG/TH	Rabbit anti-FG (1:1000, AB153-I, Millipore, Hayward, CA, USA)<br>Mouse anti-TH (1:200, T2928, Sigma-Aldrich, St. Louis, MO, USA)<br>24 h, room temperature (RT)	Alexa 594 donkey anti-rabbit IgG (1:500, A21207, Invitrogen, Carlsbad, CA, USA)<br>Alexa 647 donkey anti-mouse IgG (1:500, A31571, Invitrogen)<br>6 h, RT
Fig. 1G	TH/NeuN	Rabbit anti-TH (1:200, AB152, Millipore)<br>Mouse anti-NeuN (1:500, ab104224, Abcam, Cambridge, MA, USA)<br>24 h, RT	Alexa 488 donkey anti-rabbit IgG (1:500, A21206, Invitrogen)<br>Alexa 647 donkey anti-mouse IgG<br>6 h, RT
Fig. 3A	TH/β-actin	Rabbit anti-TH (1:1000)<br>Mouse anti-β-actin (1:2000, A5441, Sigma-Aldrich)<br>Overnight, 4 °C	HRP-conjugated anti-rabbit antibody (1:5000; ZB-2301, ZSGB-BIO, Beijing, China)<br>HRP-conjugated anti-mouse antibody (1:5000; ZB-2305)<br>1 h, RT
Fig. 3B	GAD67/β-actin	Mouse anti-GAD67 (1:1000; MAB5406, Millipore)<br>Mouse anti-β-actin<br>Overnight, 4 °C	HRP-conjugated anti-mouse antibody<br>1 h, RT
Fig. 3C-E and S4A-C	FOS/TH	Mouse anti-FOS (1:500, ab11959, Abcam)<br>Rabbit anti-TH<br>48 h, RT	Alexa 594 donkey anti-mouse IgG (1:500, A21203, Invitrogen)<br>Alexa 488 donkey anti-rabbit IgG<br>6 h, RT
Fig. 4B	Biocytin	Alexa 488-avidin (1:500, S11223, Invitrogen)<br>6 h, RT	
Fig. 5C	CRE	Rabbit anti-CRE (1:500, ab190177, Abcam)<br>24 h, RT	Alexa 488 donkey anti-rabbit IgG<br>6 h, RT

Fig. 5D	FOS	Mouse anti-FOS<br>48 h, RT	Alexa 488 donkey anti-mouse IgG<br>(1:500, A21202, Invitrogen)<br>6 h, RT
Fig. 6C	NeuN	Mouse anti-NeuN<br>24 h, RT	Alexa 594 donkey anti-mouse IgG<br>6 h, RT
Fig. 7C	TH	Mouse anti-TH<br>24 h, RT	Alexa 488 donkey anti-mouse IgG<br>6 h, RT
Fig. 9A<br>and E	CaMKII	Rabbit anti- CaMKII<br>(1:300, 13730-1-AP, Proteintech, Chicago, IL,<br>USA)<br>Sheep-a-Dig-a-POD<br>(1:150, 11633716001, Roche, Basel, Switzerland)<br>24 h, RT	Alexa 488 donkey anti-rabbit IgG<br>Alexa 594-avidin<br>(1:500, S11227, Invitrogen)<br>6 h, RT
Fig. 9C<br>and G	GFP	Rabbit anti-GFP<br>(1:200, PC408, Sigma-Aldrich)<br>Sheep-a-Dig-a-POD<br>24 h, RT	Alexa 488 donkey anti-rabbit IgG<br>Alexa 594-avidin<br>6 h, RT
Fig. 9I	D1R/β-actin	Rabbit anti-D1R<br>(1:1000, abs120388, absin, Shanghai, China)<br>Mouse anti-β-actin<br>Overnight, 4 °C	HRP-conjugated anti-rabbit antibody<br>1 h, RT<br>HRP-conjugated anti-mouse antibody<br>1 h, RT
	D2R/β-actin	Rabbit anti-D2R<br>(1:1000, abs102864, absin)<br>Mouse anti-β-actin<br>Overnight, 4 °C	HRP-conjugated anti-rabbit antibody<br>1 h, RT<br>HRP-conjugated anti-mouse antibody<br>1 h, RT
Fig. S2	FG	Rabbit anti-FG	Alexa 647 donkey anti-rabbit IgG<br>(1:500, A31573, Invitrogen)<br>6 h, RT
Fig. S9A	tdTomato	rabbit anti-RFP<br>(1:200, ab62341, Abcam)<br>Sheep-a-Dig-a-POD<br>24 h, RT	Alexa 594 donkey anti-rabbit IgG<br>Alexa 488-avidin<br>6 h, RT
Fig. S9B	EGFP	Rabbit anti-GFP<br>Sheep-a-Dig-a-POD<br>24 h, RT	Alexa 488 donkey anti-rabbit IgG<br>Alexa 594-avidin<br>6 h, RT
